# Chromosome 9p21 SNPs Associated with Multiple Disease Phenotypes Correlate with *ANRIL* Expression

**DOI:** 10.1371/journal.pgen.1000899

**Published:** 2010-04-08

**Authors:** Michael S. Cunnington, Mauro Santibanez Koref, Bongani M. Mayosi, John Burn, Bernard Keavney

**Affiliations:** 1 Institute of Human Genetics, Newcastle University, Newcastle upon Tyne, United Kingdom; 2 Department of Medicine (BMM), University of Cape Town, Cape Town, South Africa; Georgia Institute of Technology, United States of America

## Abstract

Single nucleotide polymorphisms (SNPs) on chromosome 9p21 are associated with coronary artery disease, diabetes, and multiple cancers. Risk SNPs are mainly non-coding, suggesting that they influence expression and may act in *cis*. We examined the association between 56 SNPs in this region and peripheral blood expression of the three nearest genes *CDKN2A*, *CDKN2B*, and *ANRIL* using total and allelic expression in two populations of healthy volunteers: 177 British Caucasians and 310 mixed-ancestry South Africans. Total expression of the three genes was correlated (P<0.05), suggesting that they are co-regulated. SNP associations mapped by allelic and total expression were similar (r = 0.97, P = 4.8×10^−99^), but the power to detect effects was greater for allelic expression. The proportion of expression variance attributable to *cis*-acting effects was 8% for *CDKN2A*, 5% for *CDKN2B*, and 20% for *ANRIL*. SNP associations were similar in the two populations (r = 0.94, P = 10^−72^). Multiple SNPs were independently associated with expression of each gene (P<0.05 after correction for multiple testing), suggesting that several sites may modulate disease susceptibility. Individual SNPs correlated with changes in expression up to 1.4-fold for *CDKN2A*, 1.3-fold for *CDKN2B*, and 2-fold for *ANRIL*. Risk SNPs for coronary disease, stroke, diabetes, melanoma, and glioma were all associated with allelic expression of *ANRIL* (all P<0.05 after correction for multiple testing), while association with the other two genes was only detectable for some risk SNPs. SNPs had an inverse effect on *ANRIL* and *CDKN2B* expression, supporting a role of antisense transcription in *CDKN2B* regulation. Our study suggests that modulation of *ANRIL* expression mediates susceptibility to several important human diseases.

## Author Summary

Genetic variants on chromosome 9p21 have been associated with several important diseases including coronary artery disease, diabetes, and multiple cancers. Most of the risk variants in this region do not alter any protein sequence and are therefore likely to act by influencing the expression of nearby genes. We investigated whether chromosome 9p21 variants are correlated with expression of the three nearest genes (*CDKN2A*, *CDKN2B*, and *ANRIL*) which might mediate the association with disease. Using two different techniques to study effects on expression in blood from two separate populations of healthy volunteers, we show that variants associated with disease are all correlated with *ANRIL* expression, but associations with the other two genes are weaker and less consistent. Multiple genetic variants are independently associated with expression of all three genes. Although total expression levels of *CDKN2A*, *CDKN2B*, and *ANRIL* are positively correlated, individual genetic variants influence *ANRIL* and *CDKN2B* expression in opposite directions, suggesting a possible role of *ANRIL* in *CDKN2B* regulation. Our study suggests that modulation of *ANRIL* expression mediates susceptibility to several important human diseases.

## Introduction

**Figure 1:**
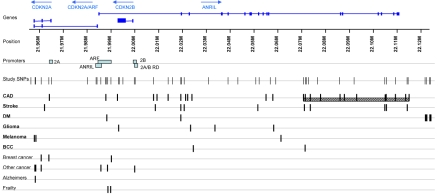
SNPs associated with disease in the chromosome 9p21.3 region. Genes are illustrated in blue at the top, with arrows representing the direction of transcription. SNPs typed in our study and SNPs associated with various diseases are represented by black bars. Diseases in bold are those with association data from genomewide association studies. The hatched box represents the core risk haplotype for CAD defined by Broadbent *et al* [35]. Promoter regions for each gene are shown as pale blue boxes. DM = diabetes mellitus type II; BCC = basal cell carcinoma.

The chromosome 9p21.3 region adjacent to the loci encoding the cyclin-dependent kinase inhibitors *CDKN2A* (ENSG00000147889) and *CDKN2B* (ENSG00000147883) is an important susceptibility locus for several diseases with a complex genetic background. Recent genome-wide association (GWA) studies have shown that single nucleotide polymorphisms (SNPs) in this region are associated with coronary artery disease (CAD) [1]–[4], ischaemic stroke [5], [6], aortic aneurysm [7], type II diabetes [8],[9], glioma [10], [11], and malignant melanoma [12]. Candidate gene approaches have also reported SNPs in this region to be associated with breast [13], [14], ovarian [15], and pancreatic carcinoma [16], melanoma [17], and acute lymphoblastic leukaemia [18], as well as with poor physical function in the elderly [19]. Variants associated with these diseases are represented in Figure 1. Most of the risk variants in the chromosome 9p21 region identified by GWA studies are in non-coding regions, suggesting that their effects are likely to be mediated by influences on gene expression. Sequence variation can influence expression by *cis* or *trans* mechanisms. *Trans*-acting elements influence transcript levels of both alleles via diffusible factors and are usually located distant to the genes they regulate, whereas *cis*-acting elements act on genes on the same chromosome in an allele-specific manner and are usually located close to the genes they regulate. Since most reported risk variants in the 9p21 region do not appear in mature transcripts, and there are no known or predicted microRNAs mapping to this region [20]–[23], these variants are unlikely to produce diffusible *trans*-acting factors and are therefore likely to influence expression of nearby genes in *cis*. Genes in the region include the cyclin-dependent kinase inhibitors *CDKN2A* (*p16^INK4a^*) including its alternative reading frame (*ARF*) transcript variant (*p19^ARF^*), *CDKN2B* (*p15^INK4b^*), and a recently-discovered non-coding RNA, designated *ANRIL* (*CDKN2BAS*, ENSG00000240498), that undergoes splicing and is transcribed from the opposite strand to *CDKN2A/B*. The ARF/CDKN2A/B proteins are established tumour suppressors deleted in a range of cancers including familial cutaneous malignant melanoma [24]; they block cell cycle progression and influence key physiological processes such as replicative senescence, apoptosis, and stem-cell self-renewal [25]. *Cis*-acting regulatory elements for these genes have been identified *in vitro* using reporter assays [26]–[30], but expression levels are also influenced by factors such as age, chemotherapeutic agents, DNA damage by ultraviolet or ionizing radiation, and levels of transcriptional regulators [31], all of which are likely to act in *trans*. The function of *ANRIL* is unknown, but other processed non-coding RNAs are involved in the regulation of gene expression through transcriptional and translational control mechanisms [32].

Genetic effects on expression can be assessed by comparing total expression levels in individuals with different genotypes at a putative regulatory locus. This is termed expression quantitative trait locus (eQTL) mapping [33]. This approach utilises information from all members of the population, but reflects the net effect of both *cis* and *trans*-acting influences; the sensitivity to detect *cis*-acting effects is therefore reduced in the presence of significant variation in *trans*-acting influences such as the environmental factors outlined above. An alternative approach that is specific for mapping *cis*-acting influences is to measure allelic expression (aeQTL mapping). An unequal amount of transcript arising from each allele in an individual heterozygous for a transcribed polymorphism indicates the presence of *cis*-acting effects on expression. While traditional eQTL analysis assesses the influence of polymorphisms by comparing expression between samples, allelic expression analysis compares the expression levels of alleles within individual samples, making it much more robust to *trans*-acting influences that affect both alleles such as age, gender, population stratification, or experimental variability. This maximises the sensitivity for detecting *cis*-acting effects [34].

Variants associated with CAD span a region greater than 100kb, but the association is accounted for by SNPs within a 53kb interval that define a core risk haplotype [35]. Lead SNPs for CAD and diabetes are in separate LD blocks in Caucasians and are independently associated with the two separate diseases [35]. To date, CAD risk SNPs have shown inconsistent association with *CDKN2A*, *CDKN2B* and *ANRIL* by eQTL mapping. One CAD risk SNP was associated with altered *ANRIL* expression in blood, but not with *CDKN2A* or *CDKN2B* expression [36], whilst a different CAD risk SNP has been associated with reduced expression of all three genes in peripheral blood T-cells [37]. However, the latter study found no association with expression for other CAD risk SNPs [37], and another report also found no association of a lead CAD risk SNP with these genes or with global gene expression in primary vascular tissue and lymphoblastoid cells [38]. Based on evolutionary conservation and effects on expression, individual SNPs (rs10757278 and rs1333045) have been highlighted as potential causal variants for the association with CAD [36], [37]. However, if multiple *cis*-acting effects are present at a locus, resolving a disease association by fine-mapping may not be possible. Examining gene expression rather than disease phenotype increases the power to map *cis*-acting effects, and we used this approach to determine whether multiple sites independently influence expression. Caucasian populations have strong linkage disequilibrium (LD) in the chromosome 9p21 region which limits the ability to separate the effects of individual SNPs on expression [35]. Populations of African ancestry have less LD [39], [40], which can be exploited to improve the fine-mapping of functional polymorphisms associated with quantitative traits [41], [42].

We therefore used eQTL and aeQTL mapping to perform detailed fine-mapping of the association of SNPs at the 9p21.3 locus with expression of *CDKN2A*, *CDKN2B* and *ANRIL* using a mixed-ancestry South African (SA) population, as well as a British Caucasian cohort. We identified multiple SNPs independently associated with expression of each gene, suggesting that several sites may modulate disease susceptibility. The markers identified in GWA studies were all associated with allelic expression of *ANRIL*, but association with the other two genes was only detectable for some of them. Our study suggests that modulation of *ANRIL* expression mediates susceptibility to a range of important human diseases.

## Results

We measured expression of *CDKN2A*, *CDKN2B* and *ANRIL* in peripheral blood from 310 healthy SA individuals (demographic details provided in the Methods section). Allelic expression was assessed for each gene using two transcribed SNPs located within the same exon. We selected 56 SNPs that tag the common variation in the region, specifically including SNPs with previously reported phenotypic associations. The results of allelic expression mapping in this population were compared with conventional mapping using total expression in the same samples; and with allelic expression mapping in a separate population of 177 healthy British Caucasians. Information on the selected SNPs and genotyping data are summarised in Table S1.

### Inter-individual variation in expression

Total expression levels showed substantial inter-individual variation for each of the three genes, up to 13.9-fold for *CDKN2A*, 36.1-fold for *CDKN2B*, and 25.5-fold for *ANRIL*. Allelic expression ratios at individual transcribed markers also showed considerable inter-individual variation, up to 5.6-fold for *CDKN2A*, 2.4-fold for *CDKN2B*, and 6.8-fold for *ANRIL*. Plots of the allelic expression ratios at each transcribed SNP in the SA and Caucasian cohorts are shown in Figure S1 and Figure S2 and plots of the normalised total expression Ct values are shown in Figure S3. Standard errors for *ANRIL* were higher than for the other two genes in both the allelic and total expression assays, which is likely to be due to the fact that peripheral blood expression of *ANRIL* was lower than for *CDKN2A* and *CDKN2B*.

We estimated the proportion of the variance in total expression that can be attributed to *cis*-acting effects for each transcribed SNP in the three genes, as described in the Methods section. For *CDKNA* this proportion was 8% when rs3088440 was used to estimate the variance in cis acting effects, and 4% when rs11515 was used. For *CDKN2B* the corresponding values were 5% (using rs3217992), 5% (using rs1063192) and for *ANRIL* 20% (using rs10965215), and 19% (using rs564398).

### Correlation of *CDKN2A*, *CDKN2B*, and *ANRIL* expression

Total expression levels of *CDKN2A*, *CDKN2B* and *ANRIL* were positively correlated (r = 0.24 to 0.30, all P<4×10^−5^) as shown in Figure S4, suggesting that expression of these genes is co-regulated.

### Allelic expression versus total expression for mapping *cis*-acting effects

Allelic expression ratios (AER) measured at the two transcribed SNPs in each gene were highly correlated (*CDKN2A* r = 0.68 P = 1.7×10^−3^; *CDKN2B* r = 0.80 P = 1.7×10^−12^; *ANRIL* r = 0.90 P = 1.0×10^−26^; all genes combined r = 0.96 P = 3×10^−61^) as shown in Figure S5. This was expected since the two transcribed SNPs selected to assess AER in each gene are located in the same exon and the same transcripts. We therefore used the AERs from both transcribed markers in each gene (as described in the Methods section) for the aeQTL analysis. This increased the number of informative heterozygotes at which allelic expression could be assessed for each gene and increased the power to detect significant effects, as shown in Table 1.

**Table 1:** Increase in number of informative heterozygotes and associated SNPs using two transcribed SNPs per gene.

Transcribed SNP	Number (%) of informative heterozygotes in Caucasian cohort (n = 177)	Number (%) of informative heterozygotes in SAcohort (n = 310)	Number (%) of mapping SNPs significantly associated with AER at transcribed SNP(s) in Caucasian cohort* (n = 53 SNPs)	Number (%) of mapping SNPs significantly associated with AER at transcribed SNP(s) in SA cohort* (n = 56 SNPs)
*CDKN2A* rs3088440	23 (12%)	103 (33%)	5 (9%)	2 (4%)
*CDKN2A* rs11515	33 (18%)	75 (24%)	0 (0%)	9 (16%)
***CDKN2A*** ** markers combined**	**54 (29%)**	**159 (51%)**	**3 (6%)**	**11 (20%)**
*CDKN2B* rs3217992	71 (38%)	112 (36%)	0 (0%)	4 (7%)
*CDKN2B* rs1063192	70 (37%)	87 (28%)	0 (0%)	2 (4%)
***CDKN2B*** ** markers combined**	**90 (48%)**	**164 (53%)**	**5 (9%)**	**5 (9%)**
*ANRIL* rs10965215	70 (37%)	155 (50%)	25 (47%)	27 (48%)
*ANRIL* rs564398	67 (36%)	85 (28%)	23 (43%)	22 (39%)
***ANRIL*** ** markers combined**	**80 (43%)**	**187 (61%)**	**30 (57%)**	**31 (55%)**

*Multiple testing was taken into account by calculating the family wise error rate using a Bonferroni correction for the 56 SNPs tested. Associations with family wise error rate using a threshold of 0.05 (that corresponds to a nominal P value of 8.9×10^−4^ or −log_10_P of 3.05) were called significant. SNPs with less than 8 informative heterozygotes were excluded.

**Figure 2:**
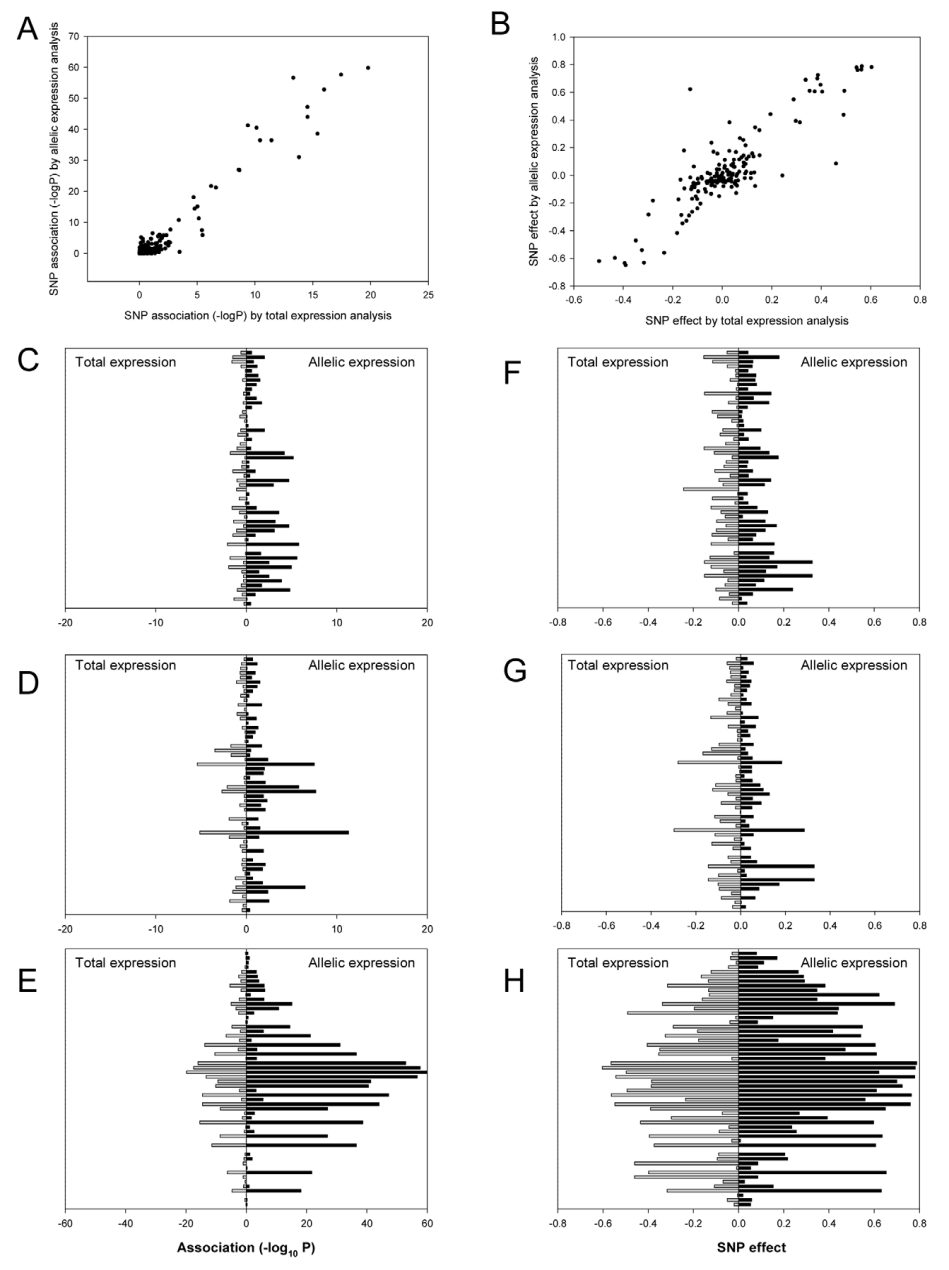
Significance of associations and effect size estimates using total and allelic expression. Scatter plots depict the P-values (A) and estimates of effect size (B) obtained for each SNP for all three genes by eQTL (X-axis) and aeQTL (Y-axis) mapping. Bar charts show the comparison of the significance of association for *CDKN2A* (C), *CDKN2B* (D), and *ANRIL* (E); and the effect size estimates for *CDKN2A* (F), *CDKN2B* (G), and *ANRIL* (H). The Y-axes on the bar charts show the 56 SNPs ordered by chromosome location (most telomeric at the top). Grey bars to the left represent total expression and black bars to the right represent allelic expression.

Unlike allelic expression ratios, total expression data may be influenced by covariates that influence expression in *trans*. We therefore corrected total expression values for covariates (age, sex, and ethnicity) and excluded outlying individuals as described in the Methods section. These corrections did not significantly alter the results of the eQTL analysis, as shown in Figure S6. All subsequent analyses are presented using the covariate-corrected eQTL data. We compared *cis*-acting effects assessed by eQTL and aeQTL mapping, as shown in Figure 2. There was a strong correlation both for the effect size (r = 0.87, P = 4.7×10^−51^) and significance of association (r = 0.97, P = 4.8×10^−99^) at each mapping SNP between the two techniques. However, the associations were more significant for allelic expression than for total expression analysis, indicating that allelic expression had greater power for detecting *cis*-acting effects. This suggests that *trans*-acting effects make a substantial contribution to the overall variance of expression in these genes, which is consistent with our estimates that *cis*-acting effects account for only between 4 and 20% of the overall variance in expression of these genes.

### Comparison of *cis*-acting effects between populations and combined analysis

**Figure 3:**
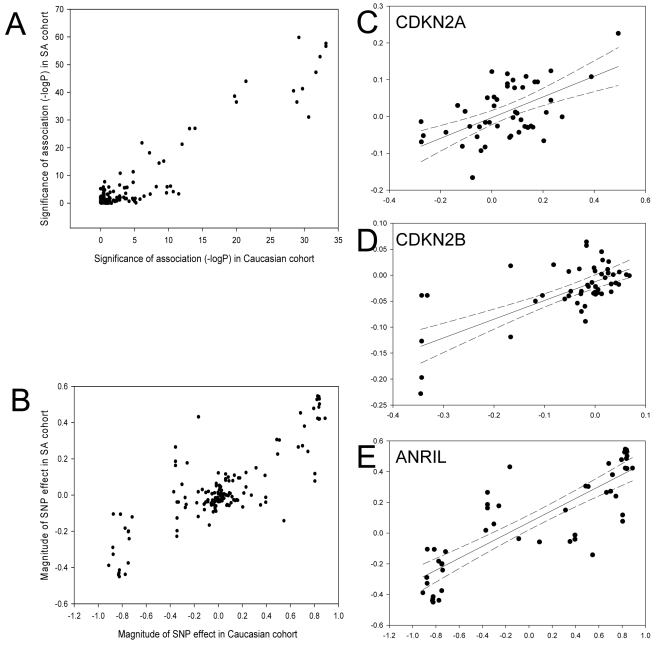
SNP effects in the SA and Caucasian cohorts. Scatter plots show the correlation between aeQTL results obtained in the SA (Y-axis) and Caucasian cohorts (X-axis) for: (A) significance of association with expression (−log P value) for all three genes; (B) effect size at each SNP for all three genes; (C) effect size at each SNP for *CDKN2A* only; (D) effect size at each SNP for *CDKN2B* only; (E) effect size at each SNP for *ANRIL* only. Linear regression line for the association is shown as a solid line with the 95% confidence intervals shown as dotted lines.

We compared aeQTL analysis between the SA and British Caucasian samples. Results of aeQTL mapping were highly correlated between the two populations, both for the significance of the detected association (r = 0.94, P = 10^−72^) and the estimated magnitude of the effect on expression for each SNP (r = 0.82, P = 2×10^−38^), as shown in Figure 3. Patterns of LD in the two populations are shown in Figure S7. Minor allele frequency in the SA population was higher (which increases the proportion of informative heterozygotes for allelic expression analysis) for 33 of the 53 SNPs typed in both populations.

**Figure 4:**
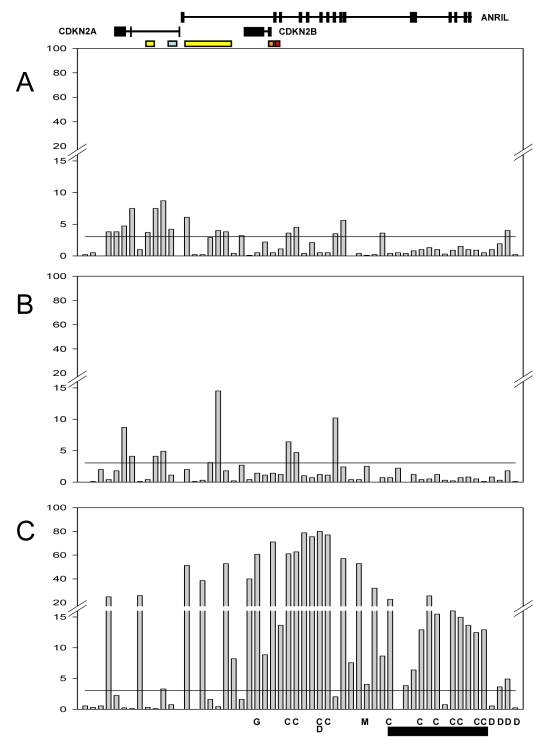
Significance of association with expression for SNPs in the combined population. The Y-axis represents the −log P value for individual SNPs (shown in chromosomal order along the X-axis) for: *CDKN2A* (A); *CDKN2B* (B); *ANRIL* (C). The horizontal black line on each graph represents the significance threshold after adjustment for multiple testing (family wise error rate of 0.05 corresponding to −log_10_P = 3.05). The relative location of genes and promoter elements is represented at the top (*CDKN2A* and *CDKN2A*/ARF promoters yellow; *ANRIL* promoter blue; *CDKN2B* promoter orange; *CDKN2A*/ARF regulatory domain red). Letters along the bottom represent associations from GWA studies (C = CAD, D = diabetes, M = melanoma, G = glioma) and the black bar at the bottom represents the core risk haplotype for CAD defined by Broadbent *et al* [35].

In view of the similarity of the effects in the two cohorts, we combined the data in subsequent analyses, increasing the power to detect *cis*-acting effects of smaller magnitude and enabling us to adjust for the effects of individual SNPs. The significance of associations for individual SNPs in the combined cohort is shown in Figure 4. Subsequent results refer to the combined dataset, with specific discussion of differences between the populations where relevant.

As described in the Methods section, we defined significance thresholds for all SNP associations using the family wise error rate (FWER) where multiple testing was taken into account by using a Bonferroni correction for the 56 SNPs tested. Associations with a FWER threshold of 0.05 (corresponding to a nominal P-value of 8.9×10^−4^, −log_10_P of 3.05, and −log_10_ FWER of 1.3) were regarded as significant. Table S2 shows the −log_10_ of the nominal P-values and FWER for all SNP associations, and nominal P-values are reported in the text.

The effect of each SNP on AER is also shown in Table S2. The maximum change in allelic expression associated with any SNP was 1.4-fold for *CDKN2A*, 1.33-fold for *CDKN2B*, and 1.97-fold for *ANRIL*. Due to the power of our combined dataset we were able to detect SNP effects on allelic expression as small as 1.05-fold that were significant.

### Multiple sites influence *CDKN2A*, *CDKN2B*, and *ANRIL* expression

**Figure 5:**
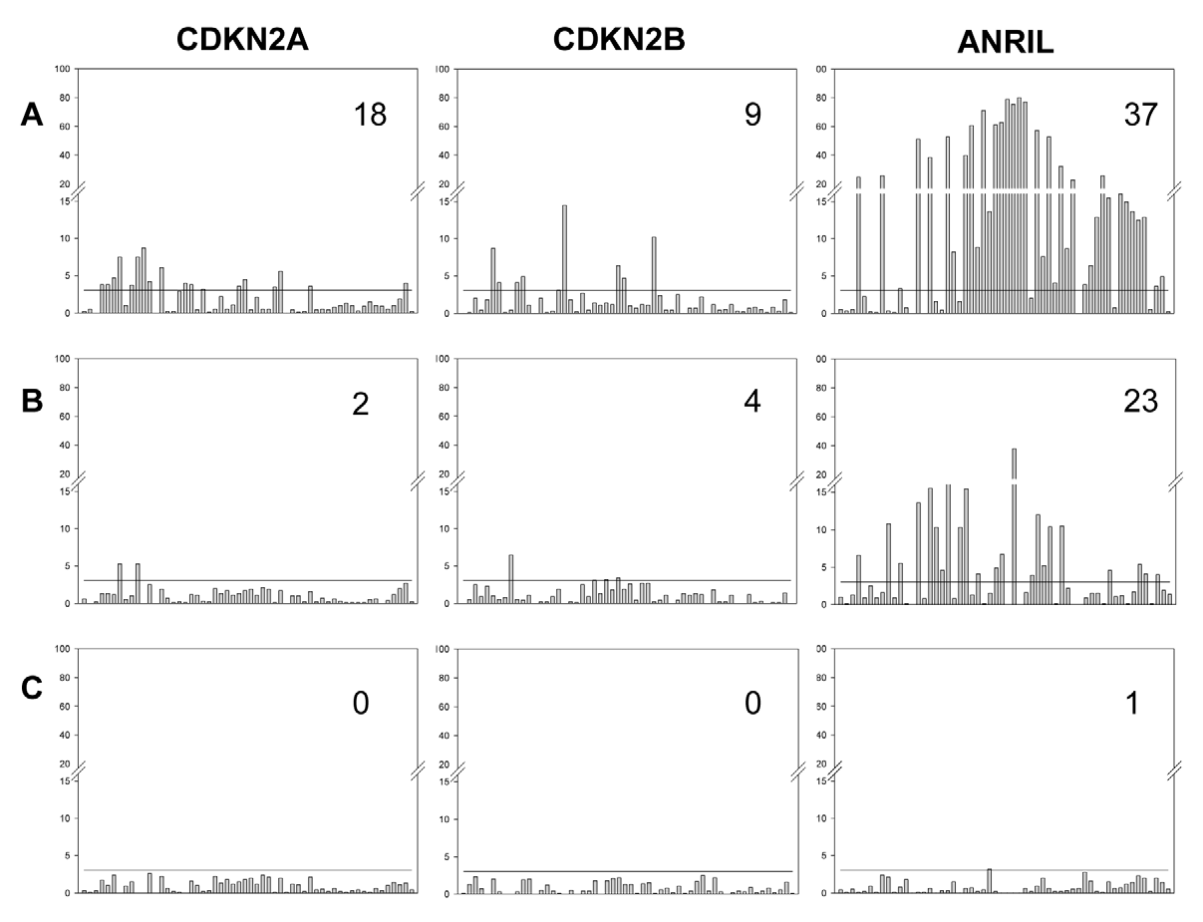
Effect of sequential adjustment for most highly associated SNPs. Bars represent the significance of association (−log_10_P) for each SNP. (A) Unadjusted. (B) Adjusted for the most highly associated SNP for each gene (*CDKN2A* rs7036656, *CDKN2B* rs3218018, *ANRIL* rs564398). (C) Adjusted for the two most highly associated SNPs for each gene (*CDKN2A* rs7036656 and rs36228834, *CDKN2B* rs3218018 and rs3814960, *ANRIL* rs564398 and rs10965215). Values in the top right corner are the number of significantly associated SNPs after each round of adjustment, following correction for multiple testing. The horizontal black line on each graph represents the significance threshold after adjustment for multiple testing (family wise error rate of 0.05 corresponding to −log_10_P = 3.05).

As shown in Figure 4, multiple SNPs were associated with *cis*-acting influences on expression of *CDKN2A*, *CDKN2B* and *ANRIL*. This could be the result of multiple independent loci influencing expression of each gene, but could also be a reflection of strong LD in the region since associations might be observed for ‘non-functional’ SNPs (that do not directly influence expression) which are in LD with other ‘functional’ polymorphisms. Adjusting for the effect of individual SNPs was used to assess whether multiple SNPs were independently correlated with expression of the three genes, as shown in Figure 5. For each gene stepwise adjustments were made for the effect of the SNP which showed the most significant association with expression, until independent effects could no longer be detected. Associations remained significant after adjusting for the top SNP for *CDKN2A* and *CDKN2B*, and the top two SNPs for *ANRIL*.

**Figure 6:**
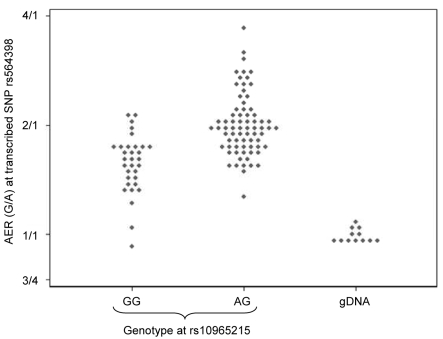
Effect of genotype at rs10965215 on allelic expression ratio of transcribed *ANRIL* SNP rs564398. Diamonds represent the allelic expression ratio for each individual, all of whom are heterozygous for the transcribed SNP rs564398. The first column shows individuals who are homozygous for rs10965215 (mean ratio 1.57), and the second column shows individuals who are heterozygous for rs10965215 (mean ratio 2.00). The third column shows the expression ratio obtained from genomic DNA in individuals who are heterozygous for the transcribed SNP rs564398, where the two alleles are present in a 1∶1 ratio (mean ratio 1.00).

Our results indicate that even after adjusting for the effects of the most significant marker, some of the remaining SNPs still showed significant association with *ANRIL* expression. This could be explained by the presence of more than one functional polymorphism affecting expression, but could also reflect the presence of a functional polymorphism that is in disequilibrium with both markers. However, examination of the allelic expression patterns provides additional support for the presence of multiple sites affecting expression. For example, Figure 6 shows the allelic expression ratios observed at the transcribed SNP rs564398 in *ANRIL*, grouped according to the genotype at rs10965215. These two SNPs are in strong LD (D′ = 0.98), hence the absence of individuals homozygous for the A allele at rs10965215 that are heterozygous at rs564398. We observe that the G allele of the transcribed SNP (rs564398) is overexpressed (G/A AER values greater than 1), however overexpression is stronger (P = 10^−15^ using the Mann-Whitney test) for individuals that are also heterozygous at the second polymorphism (rs10965215). This pattern is not consistent with allelic expression being determined by a single biallelic polymorphism acting in *cis* and suggests that there is more than one functional polymorphism or that this polymorphism is multiallelic. Such patterns were common in our data.

The direction of *cis*-acting effects on expression was compared between genes for SNPs showing significant associations with expression of each gene, as shown in Table 2. SNP effects for *CDKN2A* and *ANRIL* were in the same direction for all 10 SNPs, meaning that alleles associated with overexpression of *CDKN2A* were also associated with overexpression of *ANRIL*. By contrast, for all 8 SNPs that were significantly associated with allelic expression of both *CDKN2A* and *CDKN2B*, the alleles associated with *CDKN2A* overexpression were associated with *CDKN2B* underexpression. Similarly for all 3 SNPs significantly associated with allelic expression of both *CDKN2B* and *ANRIL*, alleles associated with overexpression of *CDKN2B* were associated with *ANRIL* underexpression. The total expression analysis had insufficient power for similar analyses to be performed.

**Table 2:** Correlation of SNP effects between genes by aeQTL mapping.

	*CDKN2A-ANRIL*	*CDKN2B-ANRIL*	*CDKN2A-CDKN2B*
**SNP effects same direction**	10	0	0
**SNP effects opposite**	0	3	8

The table shows the SNP effect between genes for SNPs that show significant association (using family wise error rate threshold 0.05) with expression of both genes. Gene pairs are shown along the top. SNP effects in the same direction means that a SNP is associated with overexpression or underexpression of both genes, whereas SNP effects in the opposite direction means that a SNP associated with underexpression of one gene is associated with overexpression of the other gene.

### *In vivo* effects of putative regulatory elements identified *in vitro*

We investigated whether SNPs within regulatory regions previously identified by *in vitro* reporter assays were associated with *cis*-acting effects on expression *in vivo*. The effect on gene expression and significance of the association for each SNP is summarised in Table S2.

*CDKN2A* expression was significantly correlated with SNPs in its promoter and the *ARF* transcript promoter [26]–[29], and with SNPs close to the RD^INK4/ARF^ domain that has been shown to regulate expression of *CDKN2A*, *ARF* and *CDKN2B in vitro* [30].

*CDKN2B* expression was also significantly correlated with SNPs in the *CDKN2A* and *ARF* promoter regions, suggesting that these elements influence expression of both genes. *CDKN2B* expression was not significantly correlated with the single SNP typed in its promoter (rs2069418) prior to adjustment, but this became significant after adjustment for the most significant SNP in the *ARF* promoter (rs3218018).

*ANRIL* expression was strongly associated with SNPs in the *CDKN2B* promoter (P = 10^−72^), *ARF* promoter (P up to10^−53^) and RD^INK4/ARF^ domain (P = 10^−12^), as well as with SNPs adjacent to the *CDKN2A* promoter (rs3731239, P = 10^−25^).

These data validate *in vivo* the function of the regulatory elements identified by *in vitro* transfection studies, and confirm that shared *cis*-acting elements influence expression of *CDKN2A*, *CDKN2B* and *ANRIL*.

### CAD, diabetes, and cancer risk variants are associated with *cis*-acting effects on expression

We examined the correlation of allelic expression of *CDKN2A*, *CDKN2B* and *ANRIL* with SNPs reported to confer disease susceptibility. The effect on gene expression and significance of the association for each SNP is summarised in Table 3.

**Table 3:** Effect size and significance of association for SNPs associated with disease.

SNP	Reported phenotypic associations	Risk allele	Minor allele	*CDKN2A* effect (fold change)	*CDKN2A* −log_10_P	*CDKN2A* −log_10_ FWER	*CDKN2B* effect (fold change)	*CDKN2B* −log_10_P	*CDKN2B* −log_10_ FWER	*ANRIL* effect (fold change)	*ANRIL* −log_10_P	*ANRIL* −log_10_ FWER
**rs3731257**	Ovarian ca	G	A	0.883	3.8	2.1	1.011	0.4	0	0.596	24.1	22.4
**rs3088440**	Melanoma, pancreatic ca, ovarian ca, bladder ca	A	A	0.919	3.8	2.1	1.050	1.8	0.1	0.828	2.1	0.4
**rs11515**	Alzheimers, bladder ca, pancreatic ca	C	G	1.084	4.7	3.0	0.880	8.7	7.0	0.968	0.2	0
**rs3731249**	Breast ca, melanoma, ALL	T	T	1.404	7.5	5.8	0.752	4.1	2.4	0.899	0.1	0
**rs3731239**	CAD, breast ca	A	C	0.947	1	0	0.997	0.1	0	1.652	25.2	23.5
**rs2811712**	Frailty, breast ca	A	G	1.079	2.9	1.2	0.938	3.1	1.4	1.155	1.5	0
**rs3218018**	Diabetes	G	G	1.108	4	2.3	0.805	14.5	12.8	1.097	0.4	0
**rs3218009**	CAD	G	C	1.059	0.4	0	0.990	0.2	0	1.659	7.7	6.0
**rs3218005**	Breast ca	C	C	1.087	3.2	1.5	0.940	2.7	1.0	1.163	1.5	0
**rs3217992**	CAD	A	A	0.990	0.1	0	1.009	0.4	0	0.579	39.7	38.0
**rs1063192**	Glioma	C	C	1.036	0.5	0	0.976	1.4	0	1.829	61.3	59.6
**rs7044859**	CAD, stroke	A	T	1.089	3.6	1.9	0.945	6.4	4.7	1.797	61.7	60.0
**rs496892**	CAD, stroke	G	A	1.102	4.5	2.8	0.952	4.7	3.0	1.775	63.3	61.6
**rs564398**	Diabetes, CAD, stroke	A	G	1.034	0.5	0	0.978	1.2	0	1.865	81.3	79.6
**rs7865618**	CAD, stroke	A	G	1.035	0.5	0	0.979	1.1	0	1.870	78.2	76.5
**rs1011970**	Melanoma	T	T	0.995	0.1	0	0.953	2.5	0.8	0.802	3.8	2.1
**rs10116277**	CAD, stroke	T	G	0.979	0.4	0	0.984	0.7	0	1.573	21.9	20.2
**rs1333040**	CAD, stroke	T	C	0.963	1	0	1.012	0.4	0	1.406	12.1	10.4
**rs10757274**	CAD	G	G	1.039	1	0	0.975	1.2	0	0.685	14.5	12.8
**rs2383206**	CAD, stroke	G	A	0.968	0.9	0	1.007	0.2	0	1.456	15	13.3
**rs2383207**	CAD, stroke	G	A	0.946	1.5	0	0.982	0.7	0	1.468	14	12.3
**rs1333045**	CAD	C	C	1.037	1	0	0.981	0.8	0	0.700	12.8	11.1
**rs10757278**	CAD, stroke	G	G	1.039	0.9	0	0.986	0.5	0	0.700	11.7	10.0
**rs1333049**	CAD	C	C	1.023	0.5	0	0.996	0.1	0	0.704	12.1	10.4
**rs2891169**	Diabetes	G	A	1.042	1	0	0.977	0.8	0	1.059	0.5	0
**rs2383208**	Diabetes	G	G	1.081	1.9	0.2	0.989	0.3	0	1.239	3.4	1.7
**rs10811661**	Diabetes	T	C	1.182	4	2.3	0.957	1.8	0.1	1.339	4.6	2.9
**rs10757283**	Diabetes	T	T	1.010	0.2	0	1.002	0.1	0	1.024	0.2	0

Data shown are for aeQTL mapping in the combined population. Effects are reported as fold changes in expression for individuals who are homozygous for the minor allele relative to individuals who are homozygous for the major allele (calculated from allelic expression data using two transcribed SNPs per gene). Association for each SNP is presented as the −log_10_ P-value and the −log_10_ of the family wise error rate (FWER) using a Bonferroni correction for the 56 SNPs tested. Associations that were significant using a FWER threshold of 0.05 (corresponding to −log_10_P of 3.05, or −log_10_FWER of 1.3) were regarded as significant.

#### CAD and stroke

SNPs within the core risk haplotype region for CAD [35] were associated with *ANRIL* expression (P up to 10^−21^), but none were associated with *CDKN2A* or *CDKN2B* expression. CAD risk alleles were all associated with reduced *ANRIL* expression, up to 1.9-fold, suggesting that expression of *ANRIL*, rather than *CDKN2A* or *CDKN2B*, might mediate atherosclerosis susceptibility. However, other CAD risk variants located telomeric to the core risk haplotype region such as rs7044859 and rs496892 showed substantially larger effects and stronger associations with *ANRIL* expression (P<10^−60^ for each SNP), and were also significantly associated with *CDKN2A* and *CDKN2B* expression (P<10^−4^ for each SNP). The CAD risk alleles at these SNPs correlated with reduced expression of *ANRIL* and *CDKN2A*, but increased *CDKN2B* expression. Associations for these SNPs remained significant after adjusting for the effect of the lead CAD SNPs within the core risk haplotype region (rs10757274, rs2383206, rs10757278 and rs1333049) [35], but SNPs within the core risk haplotype were no longer significantly associated with *ANRIL* expression after adjusting for the effect of SNPs at the distal locus (rs10965215 and rs564398). This suggests that the core CAD risk haplotype does not account for all of the observed association with *ANRIL* expression in peripheral blood.

Based on evolutionary conservation and effects on *ANRIL* transcription, rs1333045 within the core risk haplotype has been previously highlighted as a potential functional variant responsible for conferring susceptibility to CAD at the 9p21 locus [36]. In our analysis rs1333045 was associated with *ANRIL* expression (P = 10^−12^), but not with *CDKN2A* or *CDKN2B* expression. Its effects were similar to those of other SNPs in the core risk haplotype for CAD. After adjusting for the effect of rs1333045, 32 SNPs remained significantly associated with *ANRIL* expression, suggesting that the effect attributed to such variants was not due to LD with rs1333045.

#### Diabetes

The lead chromosome 9p21 SNPs associated with diabetes in GWA studies are located in a separate LD block to the CAD risk variants [7], [9], and the phenotypic effects of CAD and diabetes variants have been shown to be independent [35]. Diabetes risk alleles in this region (rs10811661-T and rs2383208-A) were associated with under-expression of *ANRIL*, but were not associated with *CDKN2A* or *CDKN2B* expression in our Caucasian population. However, these SNPs showed no association with expression of *ANRIL* in the SA population, despite greater power to detect effects in this cohort.

A separate locus for diabetes susceptibility in the chromosome 9p21 region in Caucasians is located within the region associated with CAD risk. The rs564398-T risk allele at this locus is associated with diabetes [8], CAD [35] and stroke [5]. This SNP had the strongest association with *ANRIL* expression of all the SNPs we tested (P = 10^−81^), but was not significantly associated with *CDKN2A* or *CDKN2B* expression. The rs564398-T risk allele was associated with *ANRIL* underexpression, and the association remained significant after adjusting for the effect of rs10811661, the lead diabetes SNP. However, the association with rs10811661 was no longer significant after adjustment for rs564398.

#### Cancers and frailty

GWA studies have recently identified chromosome 9p21 SNPs correlated with susceptibility for glioma [10], [11] and malignant melanoma [12]. The glioma risk allele rs1063192-C was highly correlated with increased *ANRIL* expression (P = 10^−61^), while the melanoma risk variant rs1011970-T correlated with reduced expression of *ANRIL*. Neither was associated with *CDKN2A or CDKN2B* expression.

Multiple candidate gene association studies have reported associations between SNPs in this region and susceptibility to a variety of diseases. These have mostly involved cancer phenotypes because the cell-cycle regulators *CDKN2A* and *CDKN2B* are recognised to be involved in predisposition to certain cancers. Such association studies have implicated 9p21 SNPs as being potentially involved in the development or therapeutic response to pancreatic [16], [43], breast [13], [14], [44], ovarian [15], [45], and bladder [46] carcinoma, as well as acute lymphoblastic leukaemia [18], and melanoma [17], [47], [48]. The SNPs associated with these phenotypes showed a significant correlation with allelic expression of one or more of the genes we examined, as summarised in Table 3. A SNP (rs2811712) that is associated with severely limited physical function in older people [19] was significantly associated with *CDKN2B* expression, but not with *ANRIL* expression.

## Discussion

This is the most detailed study to date of *cis*-acting influences on expression at the chromosome 9p21 locus. We have shown that multiple sites in the 9p21 region independently influence *CDKN2A*, *CDKN2B* and *ANRIL* expression, and demonstrated that SNPs associated with diseases including CAD, diabetes, and cancers are all highly associated with *ANRIL* expression, suggesting that modulation of *ANRIL* expression may mediate disease susceptibility. We also report novel methodology for allelic expression analysis that allowed us to combine data from multiple transcribed polymorphisms and to adjust for the effects of particular SNPs. We have demonstrated that this approach has greater power than total expression analysis for mapping *cis*-acting effects.

Total expression levels of *CDKN2A*, *CDKN2B* and *ANRIL*, which reflect the combined influence of *cis* and *trans*-acting factors, were positively correlated. This corroborates other recent data [37], and suggests that expression of these genes is co-regulated. We have shown that *trans*-acting influences account for the majority of the observed variance in expression of these genes (80–96%), and the correlation in total expression levels is likely to reflect co-regulation of the genes through *trans*-acting factors. In addition, our allelic expression analysis demonstrated that expression is also influenced by shared *cis*-acting elements in the region. Despite the positive correlation in total expression levels, *cis*-acting effects associated with individual SNP alleles may act in opposite directions; the effect of individual SNPs on *CDKN2B* expression were opposite to effects on *CDKN2A* and *ANRIL* expression (which were concordant) in our study. Because *cis*-acting effects represent only a small proportion of the overall variance in expression of these genes, the effects acting in *trans* are likely to account for the positive correlation seen in total expression, but this does not diminish the potential biological significance of the *cis*-acting effects. *ANRIL* overlaps and is transcribed in antisense with respect to *CDKN2B* [49]. It is modestly conserved across species [36] and its function is not known, but recent work has demonstrated that antisense transcription from *CDKN2B* downregulates *CDKN2B* expression in *cis* through heterochromatin formation [50]. This is consistent with our observation of an inverse effect of SNPs on *ANRIL* and *CDKN2B* expression. By contrast, *CDKN2A* and *ANRIL* showed positive correlations for both allelic and total expression in our study. *CDKN2A* and *ANRIL* do not overlap, but are transcribed divergently from transcription start sites separated by just 300 base pairs. Although the *ANRIL* promoter is currently not characterised, it may share promoter elements with *CDKN2A* and the resulting co-regulation could account for the positive correlation in expression we observed for these genes, similar to that described at other sites [51]. In this context, inhibition of *CDKN2B* expression by *ANRIL* would enable a level of crosstalk between *CDKN2A* and *CDKN2B* expression, which would be consistent with the inverse *cis*-acting effect of SNPs on *CDKN2A* and *CDKN2B* that we observed. The observation that *cis*-acting genetic effects played a greater role in expression of *ANRIL* compared to *CDKN2A* and *CDKN2B* (20% compared to less than 8% and 5% respectively) makes it a good candidate for genetic causation mediated through influences on expression.

We compared total expression and allelic expression for the investigation of *cis*-acting influences on expression. While traditional eQTL analysis assesses the influences of polymorphisms by comparing expression between samples, allelic expression analysis compares the expression levels of alleles within individual samples, making it more robust to influences that affect both alleles such as age, gender or population stratification. This offers an important advantage for dissecting such *cis*-acting influences on expression, which although of lesser magnitude than *trans*-acting influences, may be of biological importance and possibly account for the genetic susceptibility observed in recent GWA studies. For aeQTL mapping we used a novel adaptation of our previously reported methodology [52] to combine multiple transcribed SNPs per gene, which increased the number of informative individuals and the power for detecting *cis*-acting effects. We demonstrated this approach using two transcribed polymorphisms per gene, but our methodology offers the potential for the inclusion of multiple additional transcribed variants. The results obtained by eQTL and aeQTL mapping were similar, consistent with previous work suggesting that the two approaches identify the same *cis*-acting loci [42]. However, we demonstrated that aeQTL analysis had substantially greater power than the eQTL approach. Adjusting for *trans*-acting covariates including age, sex and ethnicity in our eQTL analysis did not substantially alter the results. An influence of age on *CDNK2A* has been reported [53], but there was little variability in the age of our SA cohort (90% of whom were between the ages of 18 and 30 years). The fact that allelic expression is a more efficient way to identify *cis*-acting influences on expression has implications for future studies investigating the effects of SNPs on expression at other loci, for example for the hundreds of non-coding SNPs correlated with different diseases by recent GWA studies [54].

Allelic expression quantifies the relative contributions of each allele to the mRNA pool irrespective of the absolute mRNA levels, and therefore provides information about transcriptional effects and polymorphisms within the transcript influencing RNA degradation in *cis*. By contrast, total expression analyses that quantify absolute mRNA levels are also sensitive to post-transcriptional regulatory effects, such as mRNA degradation by microRNAs. In extreme cases tight post-transcriptional regulation could keep total mRNA levels constant irrespective of the contributions of each allele to the total mRNA pool. The fact that the results of eQTL and aeQTL mapping were so similar in our study suggests that the effect of regulation at the post-transcriptional level is limited, although regulation of *CDKN2A* expression by a microRNA has been described [55]. In general, although allelic expression is a robust method for mapping sites influencing expression in *cis*, investigation of total expression and other intermediate phenotypes such as protein levels or protein activity will provide complementary information that contributes to fully understanding the phenotypic effects of *cis*-acting polymorphisms. It would be desirable to determine whether the significant associations with mRNA expression observed for *CDKN2A* and *CDKN2B* are confirmed at the protein level.

Although we had hoped to use trans-ethnic fine-mapping to refine the associations with expression, the results of aeQTL mapping were in fact very similar in the SA and Caucasian populations. This replication in a separate cohort strongly supports the validity of our findings and enabled us to perform a combined analysis of the two cohorts. This approach of pooling data from ethnically-divergent populations has been previously shown to increase the power to detect influences on expression that are shared across populations [42], [56]. The principal difference we identified between the two populations was for the SNPs associated with type II diabetes. The lead diabetes SNP rs10811661 was correlated with *ANRIL* underexpression in the Caucasian cohort, but not in the SA population, despite greater power to detect effects in that cohort. This may reflect differences in LD between the populations, but suggests that rs10811661 may not itself be the causal variant influencing diabetes susceptibility through effects on *ANRIL* expression. Studies to determine whether this SNP is associated with diabetes in populations of African origin would be of interest.

The power of our analyses to detect differences in expression enabled us to adjust for the effects of individual SNPs. Using this we were able to demonstrate that expression, and therefore probably disease predisposition, is independently influenced by multiple sites and that the observed effects cannot be explained by a single polymorphic site. From our analysis we cannot exclude the existence of rare variants with large effects, but previous resequencing studies in this region did not find rare variants associated with disease phenotypes [2], [3]. We are unable to say whether the individual SNPs for which we found associations are the actual ‘causal’ variants responsible for the effects on expression, or if the association simply reflects linkage disequilibrium between these SNPs and the causative polymorphisms. Although fine mapping studies often purport to identify causal variants, in the context of complex diseases characterising the pathways involved in disease predisposition may be more important. This is of particular interest for these genes where variation in expression is mostly due to *trans* effects which may be substantially influenced by non-genetic factors, raising the prospect that it may be amenable to therapeutic modulation. The putative causal variants rs10757278 and rs1333045 previously associated with altered *ANRIL* expression [36], [37] were significantly associated with reduced *ANRIL* expression *in vivo* in our analysis, but their effects were relatively modest compared to other SNPs in the region and adjustment for the effect of these SNPs accounted for only a small proportion of the effect observed at other SNPs. The maximum changes in expression associated with individual SNPs were substantial, up to 2-fold for *ANRIL*, but we were also able to detect effects of much smaller magnitude; the minimum significant effect was associated with just a 1.05-fold change in expression. Although the associations of SNPs with expression that we observed were statistically highly significant, we cannot say what impact such effects on expression have on disease risk. However, even small differences in gene expression due to genetic factors that are present throughout an individual's lifetime could contribute to differences in common late-onset phenotypes such as CAD and diabetes, and the effects may be even greater in tissues related to disease.

We examined *in vivo* expression in primary cells rather than in transformed cell lines. Although cell lines have been extensively used to investigate *cis*-acting influences on expression [56], [57], patterns of expression may be altered in immortalised cells, particularly for genes such as these that are associated with senescence and cell-cycle regulation. Furthermore, widely used cell lines are pauciclonal or monoclonal [58], [59] and since a significant proportion of human genes exhibit random patterns of monoallelic expression within single clones of cell lines [60], *cis*-acting effects in these cells are unlikely to be representative of polyclonal cell populations *in vivo*. Previous studies have delineated the promoters and other elements regulating *CDKN2A/ARF* and *CDKN2B* expression using reporter assays [26]–[30]. Such studies are valuable to identify causative polymorphisms, but since they examine the effects on expression outside of the normal haplotype, chromatin and cellular context their findings require confirmation by *in vivo* studies [34], [61]. Our analysis confirmed that polymorphisms in upstream regulatory elements identified by *in vitro* assays were significantly associated with *cis*-acting effects on expression *in vivo*, but we also demonstrated that other loci located up and downstream were associated with effects on expression of similar or even larger magnitude. These data highlight the complexity and multiplicity of sites influencing expression in the region. The assays we used to investigate *CDKN2A* expression also included the *ARF* transcript variant. This gave the possibility to detect sites influencing expression of both transcripts, and we were able to detect effects of SNPs in both the *CDKN2A* and *ARF* promoter regions, although differential effects of loci on individual transcripts cannot be distinguished using this approach.

All of the SNPs in the region associated with disease in GWA studies were associated with influences on *ANRIL* expression, suggesting that modulation of *ANRIL* expression may mediate susceptibility to these phenotypes. SNPs in the CAD core risk haplotype region [35] that are most strongly associated with CAD in GWA studies were associated with reduced *ANRIL* expression, but other SNPs associated with CAD which lie outside of the core risk haplotype showed independent and stronger associations with *ANRIL* underexpression. This may reflect differences in the relative importance of particular sites in the tissues responsible for the association with CAD. Indeed, the patterns of association we have observed in peripheral blood in healthy individuals may differ from those in primary disease tissues. Similarly, differences in the relative contribution of each SNP to modulation of expression in the tissues crucial for the pathogenesis of the different conditions could explain why particular diseases are associated with different subsets of SNPs that influence *ANRIL* expression. Recent work also suggests that *ANRIL* has multiple transcripts, which may be differentially expressed between tissues [36], [38]. Confirmation of our findings in tissues relevant to each disease and for different *ANRIL* transcripts would therefore be desirable, although for CAD and other complex diseases the cell populations responsible for mediating disease susceptibility are unknown and may be inaccessible. Although tissue specificity of *cis*-acting influences is well documented, variation in *cis*-acting effects is primarily explained by genetic variation, with allele-specific expression at most SNPs being the same between tissues in the same individual [62]. Analysis of expression in blood is therefore likely to give biologically relevant information despite the fact that this may not be the tissue in which influences on expression actually mediate disease susceptibility.

Previous genomewide expression analyses using microarrays and immortalised cell lines did not identify association of *CDKN2A* and *CDKN2B* expression with markers in this region, although they did not examine *ANRIL* expression [56], [57]. However, two recent studies specifically examining expression in the chromosome 9p21 region in primary cells reported associations between CAD risk SNPs and gene expression in blood [36], [37]. Jarinova *et al* found significant association of CAD risk variant rs1333045-C with *ANRIL* expression, but not with *CDKN2A* or *CDKN2B* expression [36]. Liu *et al* reported that a different CAD risk allele rs10757278-G was associated with reduced expression levels of *CDKN2A*, *CDKN2B*, and *ANRIL*, but in the same study found no correlation for five other SNPs tested, including two additional SNPs associated with CAD (rs518394 and rs564398). They also found no association for two SNPs associated with diabetes (rs10811661 and rs564398), the frailty risk SNP rs2811712, and a melanoma risk SNP (rs11515) [37]. We demonstrated that CAD risk SNPs rs1333045-C and rs10757278-G both correlate with *ANRIL* underexpression, but found no correlation of these SNPs with *CDKN2A* or *CDKN2B* expression. However, we identified highly significant influences on expression associated with other SNPs for which Liu *et al* found no association (rs10811661 with *CDKN2A* and *ANRIL*; rs564398 with *ANRIL*; rs2811712 with *CDKN2B*; rs11515 with *CDKN2A* and *CDKN2B*). These findings are likely to reflect the greater power of our analysis for detection of *cis*-acting effects due to the larger sample size and increased sensitivity of our aeQTL mapping approach.

The finding that disease associated SNPs are all associated with *ANRIL* expression suggests that *ANRIL* plays a role in influencing disease susceptibility. Although little is known about the targets of *ANRIL*, its effects may be mediated through antisense transcription regulation of *CDKN2B* in the tissues critical for the pathogenesis of the different diseases. The observation that the effects of sequence variants acting in *cis* were stronger for *ANRIL* than for *CDKN2B* may reflect selection pressure against variants that have substantial direct effects on the expression of critical genes. *CDKN2A*, *ARF* and *CDKN2B* are cell cycle regulators and are plausible candidates for involvement in the pathogenesis of the diseases for which we found SNP associations with *ANRIL*. Mutations involving these genes are well documented in glioma [63], [64] and melanoma [49], [65], [66]. Overexpression of *CDKN2A* and *CDKN2B* in murine models is associated with pancreatic islet hypoplasia and diabetes [67], [68], and there is also emerging evidence that vascular cell senescence involving these pathways is involved in the pathogenesis of atherosclerosis [69], [70].

Our data show that multiple independent sites in the chromosome 9p21 region influence *CDKN2A*, *CDKN2B* and *ANRIL* expression. SNPs associated with disease in GWA studies are all associated with *ANRIL* expression, indicating that modulation of *ANRIL* expression mediates susceptibility to a variety of conditions.

## Methods

### Participants

Peripheral blood for DNA and RNA analysis was collected from anonymous adult volunteers in two cohorts: 310 SA blood donors and 177 British Caucasians from north-east England. The self-reported ethnicity of the SA cohort was: 200 Cape mixed-ancestry; 67 African black; 19 Indian; 10 white; 4 other/unknown. 42% were male, with median age 20 years (range 17–60, lower quartile 19, upper quartile 23). In the Caucasian cohort, 50% were male, with median age 63 years (range 25–101, lower quartile 51, upper quartile 69).

### Ethics statement

The study complies with the principles of the Declaration of Helsinki. Informed consent was obtained from all participants and the study was approved by the Newcastle and North Tyneside Local Research Ethics Committee and the University of Cape Town Faculty of Health Sciences Research Ethics Committee.

### DNA and RNA extraction and cDNA synthesis

For the South African samples DNA was extracted using a phenol/chloroform method from 4ml of peripheral blood in EDTA collected at the time of the RNA sample. For the British samples, DNA was obtained from the RNA solution prior to DNase treatment.

RNA was extracted from 2.5ml of peripheral blood collected using the PAXgene system (Qiagen) following the manufacturer's protocol and was DNase treated using RQ1 RNase-Free DNase (Promega). For AEI measurements, approximately 2µg of total RNA was reverse transcribed and eluted in 20µl, using SuperScript VILO cDNA Synthesis Kit (Invitrogen) for the SA samples and SuperScript III First-Strand Synthesis System for RT-PCR (Invitrogen) for the British samples. For real-time PCR measurements, 500ng of total RNA was reverse transcribed using High Capacity RNA-to-cDNA Master Mix (Applied Biosystems) and eluted in 20µl.

### Selection of transcribed SNPs for allelic expression analysis

Using the NCBI Entrez Gene database (http://www.ncbi.nlm.nih.gov/, 28/01/08), transcribed SNPs with expected heterozygosity >0.2 in the HapMap CEU population were selected as suitable candidates for assessment of allelic expression. Transcribed polymorphisms in *ANRIL*, which was not annotated in the databases at the time of the design, were identified by comparing the reported mRNA sequence [49] with NCBI dbSNP. Transcribed SNPs selected using these criteria were: rs3088440 and rs11515 in exon 3 of *CDKN2A*; rs3217992 and rs1063192 in exon 2 of *CDKN2B*; rs10965215 and rs564398 in exon 2 of *ANRIL*. The two *CDKN2A* SNPs are also present in *ARF*, allowing the assessment of *cis*-acting influences on both of these transcripts. Another SNP rs10738605 in exon 3 of *ANRIL* also satisfied these criteria but was subsequently excluded due to poor performance of the assay.

### Selection of mapping SNPs

SNPs previously reported to be associated with disease phenotypes were selected for mapping effects on expression [6], [8], [9], [13]–[19], [43], [45]–[48], [71]–[74]. Additional tag SNPs required to capture common variation in a core region of interest (Chr9:21958155–22115505) based on HapMap CEU data were also selected using HaploView 4.0 Tagger software using the following parameters: minimum minor allele frequency 0.01, pairwise tagging, r^2^ threshold >0.8. SNPs within other functionally important elements such as *CDKN2A* and *CDKN2B* promoters [26], [29], [30], [75] or a putative *ANRIL* promoter region (which we arbitrarily defined as 1kb up and downstream of the transcription start site) were selected if they were reported more than once in NCBI dbSNP, had expected heterozygosity >5%, and were associated with alteration of transcription factor binding sites (using PROMO v.3.0.2) [76], [77]. Details of included SNPs are shown in Table S1.

### Genotyping

Multiplex SNP genotyping was performed by primer extension and MALDI-TOF mass spectrometry using iPLEX Gold technology from Sequenom (Sequenom Inc, San Diego, USA). SNP assays were designed using Sequenom's RealSNP (www.RealSNP.com) and MassARRAY Assay Design v3.0 Software (multiplex details and primer sequences available in Table S4). PCR was performed using 20ng of DNA in a 10µl reaction volume for 35 cycles using standard iPLEX methodology. Spectra were analysed using MassARRAY Typer v3.4 Software (Sequenom). Spectra and plots were manually reviewed and auto-calls were adjusted if required. Positive and negative controls were included. Individual samples with low genotype call rates (<80%) and SNP assays with poor quality spectra/cluster plots were excluded. Correspondence to Hardy-Weinberg proportions was checked for each SNP.

### Measurement of allelic expression ratios

PCR primers for the selected transcribed SNPs were designed using Primer3 (v.0.4.0) software [78]. *CDKN2A* primers span exons 3–4 and include both transcribed SNPs (rs3088440 and rs11515) in the same amplicon. *ANRIL* primers span exons 1–2 and include both transcribed SNPs (rs10965215 and rs564398) in the same amplicon. For *CDKN2B*, separate primer pairs for transcribed SNPs rs1063192 and rs3217992 were designed entirely within exon 2 (due to the distance of transcribed SNPs from the exon boundary).

Quantification of the allelic expression ratio was performed by primer extension and MALDI-TOF mass spectrometry using iPLEX Gold with similar parameters to the genotyping assay. Spectra were analysed using MassARRAY Typer v3.4 Software (Sequenom) and allelic ratios were estimated as the ratios of the area under the peak representing allele 1 to that representing allele 2. Measurements were performed in four replicates using 50ng of cDNA template. Results from amplification of genomic DNA were used as an equimolar reference to normalise the cDNA values. Genomic normalisation reactions for *CDKN2B* used the same PCR primers as used for cDNA, but for *CDKN2A* and *ANRIL* (where primers were cDNA-specific) separate assays designed to be as close as possible in size and location to the cDNA primers were used. Primer sequences are shown in Table S3. For some assays the allelic ratios measured in gDNA ratios did deviate from a 1∶1 ratio, as shown in Table S4, confirming that allelic ratios in cDNA required correction for assay bias. However, as expected the gDNA ratios for each assay were relatively homogeneous with little inter-individual variability compared to cDNA ratios (Figure S1 and Figure S2). We compared the results of expression mapping using two different normalisation strategies in the SA cohort: normalising to a mean population normalisation factor versus normalising each individual's cDNA to their own gDNA ratio. There was no difference in the results obtained using these two normalisation strategies, as shown in Figure S8. The mean gDNA ratios for each assay were the same in the SA cohort and a sample of Caucasian individuals (no significant difference using a two sample t-test), and we therefore used the mean gDNA ratios for normalisation of all samples.

The appropriateness of genomic normalisation ratios and linearity of the AER response were checked by mixing PCR products from individuals homozygous for the minor and major alleles in varying ratios (8∶1, 4∶1, 1∶1, 1∶4, 1∶8) and using these as template for the allelic expression assays. These experiments confirmed that allelic expression showed a linear response and that normalisation ratios obtained using allelic expression assays on a 1∶1 mixture of alleles for each SNP correspond to normalisation ratios obtained from genomic DNA (Table S4 and Figure S9).

Allelic expression ratios for the two transcribed markers in each gene were highly correlated (*CDKN2A* r = 0.68, p = 1.7×10^−3^; *CDKN2B* r = 0.80, p = 1.7×10^−12^; *ANRIL* r = 0.90, p = 1.0×10^−26^; all genes combined r = 0.96, p = 3×10^−61^) as shown in Figure S5; we therefore used a novel approach of combining allelic ratios from the two transcribed markers in each gene to increase the number of informative heterozygotes.

### Relative quantification of total gene expression using real-time PCR

Real-time PCR reactions were performed using TaqMan gene expression gene expression probes and reagents (Applied Biosystems) and run on a 7900HT Real-Time PCR System (Applied Biosystems). Commercially available FAM-labelled TaqMan assays were used for *CDKN2A* exons 2–3 (Hs00923894_m1) and *ANRIL* exons 1–2 (Hs01390879_m1). A custom FAM-labelled assay was used for exon 2 of *CDKN2B*. Commercially available VIC-labelled TaqMan assays were used for three reference genes shown to be suitable for normalisation of expression in peripheral blood [79], [80]: *B2M* (4326319E), *GAPD* (4326317E), and *HPRT1* (4326321E). TaqMan assays are validated by the manufacturer to have close to 100% amplification efficiency and assays were selected to quantify the same transcripts as the allelic expression assays. PCR was performed according to the manufacturer's protocol using four replicates, 25ng cDNA template per reaction, and the following multiplex combinations: *CDKN2A*/*B2M*, *CDKN2B*/*GAPD*, and *ANRIL*/*HPRT1*.

Relative total expression was analysed using the comparative cycle threshold (Ct) method. Ct values for each target gene were normalised to the mean Ct value of the three reference genes [79]. Standard errors and variances of measurements for allelic and total expression analyses in the SA population are shown in Table S5.

### Statistical analyses

The association between total expression, as measured by real time PCR, and each of the SNPs was assessed using linear regression of the log transformed normalized expression values on the genotype assuming no dominance or interactions between the effects of different SNPs. The effect of including age, sex, and ethnicity as covariates, as well as excluding outlying individuals as determined by visual inspection (highlighted in Figure S4) were investigated. Self reported ethnicity was included as a categorical variable (categorised as “Cape mixed-ancestry”, “black African”, “white”, “Indian”, and “other”). These corrections made no significant difference to the results of eQTL mapping (Figure S6). All analyses were performed using the corrected data. Plots illustrating the associations between genotype and total expression for selected SNPs are shown in Figure S10.

We analysed allelic expression ratios using an extension of the approach we published previously [52]. We restrict ourselves to biallelic markers, and code one arbitrarily chosen allele as 0 and the other as 1. We designate with *g* the phase-known and with *T* the phase-unknown genotype of an individual. The latter can be ascertained through genotyping. We assume that the amount of mRNA originating from a single allele follows a lognormal distribution where the variance does not vary between different alleles. The log of the ratio between the expression levels of both alleles, *I*, can therefore be assumed to be normally distributed.

For an individual that is heterozygous for *m* transcribed polymorphisms, *m* ratios can be determined. We designate the vector of the logarithms of these ratios as 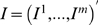. Under the assumptions above, the components of *I* are normally distributed with 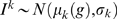 where the means 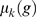 depend on the genotype 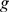 but the variance 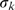 is genotype independent but may depend on the site used to measure the allelic expression ratio. We model the expected value as a linear combination of the influences of the typed polymorphisms:
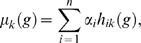
where 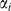 represents the effect of the *i^t^* ^h^ cis acting markers; and 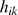 characterizes the phase between transcribed and putative *cis* acting markers:
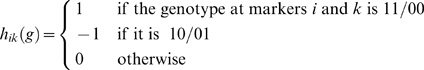
In order to assess the association between a specific SNP and allelic expression, let us consider a set of 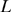 individuals. For an individual 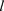 (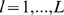) we can measure the unphased genotype 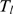 and a vector representing the log of the allelic expression ratios 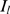. Up to a multiplicative constant the likelihood of observing a certain pattern of imbalance in this set of individuals given their genotyping results is:
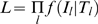
with
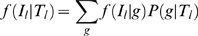
where 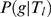 designates the probability of the phased genotypes *g* given the genotyping results 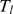 and 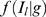 describes the density of the distribution of 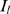 given the genotype *g*. 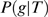 was estimated using the hap procedure from the R-package gap (as deposited in the CRAN archive http://cran.r-project.org/) to phase the genotypes of the two populations separately.

We assume that the allelic expression ratios measured at different sites are conditionally independent given the genotype. Therefore:
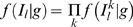
where 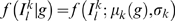 and 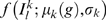 denotes the density of a normal distribution with the individual expression ratio 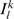 as variate, a genotype dependent mean 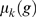 and a variance 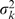. Therefore 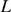 depends on 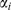 (*i* = 1,…,*n*) and 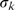 (*k* = 1,…,*m*), and maximisation of this likelihood allows assessment of the effects of single or groups of SNPs and to adjust for the effects of other markers by comparing nested models using likelihood ratio tests.

For both total and allelic expression multiple testing was taken into account by calculating the family wise error rate using a Bonferroni correction for the 56 SNPs tested. Associations with family wise error rate below a threshold of 0.05 (corresponding to a nominal P-value of 8.9×10^−4^, −log_10_P of 3.05, and −log_10_FWER of 1.3) were called significant.

From our allelic and total expression data we also estimated the proportion of total expression variance that is due to *cis*-acting effects. This assumes that *cis* and *trans*-acting factors act in an additive manner, do not interact, are independent and that there is random mating, no segregation distortion, and the locus is not subject to imprinting. Given these assumptions, we estimate the variance due to *cis* acting effects, 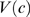, as 
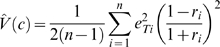
, where 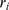 is the allelic expression ratio for individual *i*, and the proportion of the total variance due to *cis* acting effects can be estimated as 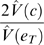, where 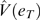 is the estimated total variance, i.e. 
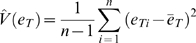
 with 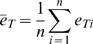 and 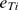 represent the total expression level for individual *i* as determined by real time PCR.

## Supporting Information

Figure S1 Allelic expression ratios at transcribed SNPs in the SA cohort. Y-axes show AER for the following transcribed SNPs: (A) *CDKN2A* rs11515; (B) CDKN2A rs3088440; (C) CDKN2B rs3217992; (D) *CDKN2B* rs1063192; (E) *ANRIL* rs564398; (F) *ANRIL* rs10965215. Each point represents an individual, with standard error bars shown. Black circles represent cDNA measurements and blue circles genomic DNA measurements.
(0.26 MB DOC)

Figure S2 Allelic expression ratios at transcribed SNPs in the Caucasian cohort. Y-axes shows AER for the following transcribed SNPs: (A) *CDKN2A* rs11515; (B) *CDKN2A* rs3088440; (C) *CDKN2B* rs3217992; (D) *CDKN2B* rs1063192; (E) *ANRIL* rs564398; (F) *ANRIL* rs10965215. Each point represents an individual, with standard error bars shown. Black circles represent cDNA measurements and blue circles represent genomic DNA measurements.
(0.21 MB DOC)

Figure S3 Total expression values in the SA cohort. Y-axes show normalised total expression Ct values relative to reference genes for: (A) *CDKN2A*; (B) *CDKN2B*; (C) *ANRIL*. Each point represents an individual, with standard error bars shown.
(0.19 MB DOC)

Figure S4 Correlations between total expression levels of *CDKN2A*, *CDKN2B* and *ANRIL*. Scatter plots show correlations between total expression levels for: (A) *CDKN2A* and *CDKN2B*; (B) *CDKN2A* and *ANRIL*; (C) *CDKN2B* and *ANRIL*. Expression values on the X- and Y-axes are shown as delta Ct values for the target gene relative to the three internal control genes. Circles represent individual samples and the crosses represent three outliers excluded from correlation analyses. Linear regression lines are shown as solid lines, with dotted lines indicating the 95% confidence intervals. Pearson correlation coefficient (r) and the P-value for each association are shown in the top left of each plot.
(0.12 MB DOC)

Figure S5 Correlation between AER in individuals heterozygous for both transcribed markers in a gene. The X- and Y-axes show the allelic expression ratio (AER) at the two transcribed SNPs in each gene. Each point represents an individual who is heterozygous for both transcribed SNPs in that gene. Circles represent *CDKN2A*, squares *CDKN2B*, and triangles *ANRIL*.
(0.03 MB DOC)

Figure S6 Effect of adjustment for covariates and outliers on total expression mapping. Scatter plots depict the estimates of effect size (A) and significance of association (B) for each of the 56 SNPs obtained using unadjusted total expression values (X-axis) versus values adjusted for covariates (age, sex, ethnicity) and with outliers removed (Y-axis). Pearson correlation coefficient (r) and the P-value for each association are shown in the top left of each plot.
(0.05 MB DOC)

Figure S7 Linkage disequilibrium in the SA and Caucasian cohorts. Figures show linkage disequilibrium between the 56 SNPs in each population: (A) D′ in Caucasian cohort; (B) D′ in SA cohort; (C) r^2^ in Caucasian cohort; (D) r^2^ in SA cohort. Colouring in (A) and (B) represents D′ values: D′ = 1, LOD<2 (blue); D′ = 1, LOD>2 (red); D′<1, LOD>2 (shades of pink); D′<1, LOD<2 (white). Shading in (C) and (D) represents r^2^ values: r^2^ = 1 (black); 0<r^2^<1 (shades of grey); r^2^ = 0 (white).
(1.05 MB DOC)

Figure S8 Effect of individual normalisation of allelic expression ratios. Scatter plots compare the estimates of effect size (A) and significance of association (B) for each of the 56 SNPs obtained using allelic expression ratios normalised to a combined normalisation factor (X-axis) versus individual normalisation of each cDNA ratio to the gDNA ratio from the same individual (Y-axis). Pearson correlation coefficient (r) and the P-value for each association are shown in the top left of each plot.
(0.05 MB DOC)

Figure S9 Linear relationship between measured and expected allelic expression ratios for alleles mixed in known ratios (8∶1, 4∶1, 1∶1, 1∶4, 1∶8) at each transcribed SNP. (A) *CDKN2A* rs3088440. (B) *CDKN2A* rs11515. (C) *CDKN2B* rs3217992. (D) *CDKN2B* rs1063192. (E) *ANRIL* rs10965215. (F) *ANRIL* rs564398.
(0.05 MB DOC)

Figure S10 Effect of genotype on total expression of *ANRIL* for selected SNPs. Y-axis shows the normalised total expression value for *ANRIL*. X-axis shows genotype for SNPs with *cis*-acting effects: (A) rs564398; (B) rs10965215; (C) rs7865618. Linear regression lines are shown as solid lines, with dotted lines indicating the 95% confidence intervals.
(0.10 MB DOC)

Table S1 Summary of included SNPs. F = SNP removed from analysis in this cohort as genotype available for <80% of individuals. CAD = coronary artery disease; MAF = minor allele frequency; HW = Hardy-Weinberg.
(0.20 MB DOC)

Table S2 Effect size and significance of association for SNPs associated with disease. Data shown are for aeQTL mapping in the combined population. Effects are reported as fold changes in expression for individuals who are homozygous for the minor allele relative to individuals who are homozygous for the major allele (calculated from allelic expression data using two transcribed SNPs per gene). Association for each SNP is presented as the −log_10_ P-value and the −log_10_ of the family wise error rate (FWER) using a Bonferroni correction for the 56 SNPs tested. Associations that were significant using a FWER threshold of 0.05 (corresponding to −log_10_P of 3.05, or −log_10_FWER of 1.3) were regarded as significant.
(0.20 MB DOC)

Table S3 Primer sequences.
(0.03 MB XLS)

Table S4 Comparison of allelic expression normalisation ratios obtained from genomic DNA and experimental equimolar mixtures.
(0.03 MB DOC)

Table S5 Comparison of variances between total expression and allelic expression measurements in the SA cohort.
(0.03 MB DOC)
