# Supplementary material for: Chromosome 9p21 SNPs Associated with Multiple Disease Phenotypes Correlate with ANRIL Expression
Source: PLoS Genet. 2010 Apr 8;6(4):e1000899. doi: 10.1371/journal.pgen.1000899 (PMC2851566; doi:10.1371/journal.pgen.1000899)
Supplement: Figure S2 — Allelic expression ratios at transcribed SNPs in the Caucasian cohort. Y-axes shows AER for the following transcribed SNPs: (A) CDKN2A rs11515; (B) CDKN2A rs3088440; (C) CDKN2B rs3217992; (D) CDKN2B rs1063192; (E) ANRIL rs564398; (F) ANRIL rs10965215. Each point represents an individual, with standard error bars shown. Black circles represent cDNA measurements and blue circles represent genomic DNA measurements. (0.21 MB DOC) [file pgen.1000899.s002.doc]

**Figure S2. Allelic expression ratios at transcribed SNPs in the Caucasian cohort.** Y-axes shows AER for the following transcribed SNPs: (A) *CDKN2A* rs11515; (B) *CDKN2A* rs3088440; (C) *CDKN2B* rs3217992; (D) *CDKN2B* rs1063192; (E) *ANRIL* rs564398; (F) *ANRIL* rs10965215. Each point represents an individual, with standard error bars shown. Black circles represent cDNA measurements and blue circles represent genomic DNA measurements.
