# Supplementary material for: Chromosome 9p21 SNPs Associated with Multiple Disease Phenotypes Correlate with ANRIL Expression
Source: PLoS Genet. 2010 Apr 8;6(4):e1000899. doi: 10.1371/journal.pgen.1000899 (PMC2851566; doi:10.1371/journal.pgen.1000899)
Supplement: Figure S3 — Total expression values in the SA cohort. Y-axes show normalised total expression Ct values relative to reference genes for: (A) CDKN2A; (B) CDKN2B; (C) ANRIL. Each point represents an individual, with standard error bars shown. (0.19 MB DOC) [file pgen.1000899.s003.doc]

**Figure S3. Total expression values in the SA cohort.** Y-axes show normalised total expression Ct values relative to reference genes for: (A) *CDKN2A*; (B) *CDKN2B*; (C) *ANRIL*. Each point represents an individual, with standard error bars shown.
