# Supplementary material for: Chromosome 9p21 SNPs Associated with Multiple Disease Phenotypes Correlate with ANRIL Expression
Source: PLoS Genet. 2010 Apr 8;6(4):e1000899. doi: 10.1371/journal.pgen.1000899 (PMC2851566; doi:10.1371/journal.pgen.1000899)
Supplement: Figure S4 — Correlations between total expression levels of CDKN2A, CDKN2B and ANRIL. Scatter plots show correlations between total expression levels for: (A) CDKN2A and CDKN2B; (B) CDKN2A and ANRIL; (C) CDKN2B and ANRIL. Expression values on the X- and Y-axes are shown as delta Ct values for the target gene relative to the three internal control genes. Circles represent individual samples and the crosses represent three outliers excluded from correlation analyses. Linear regression lines are shown as solid lines, with dotted lines indicating the 95% confidence intervals. Pearson correlation coefficient (r) and the P-value for each association are shown in the top left of each plot. (0.12 MB DOC) [file pgen.1000899.s004.doc]

**Figure S4. Correlations between total expression levels of *CDKN2A*, *CDKN2B* and *ANRIL*.** Scatter plots show correlations between total expression levels for: (A) *CDKN2A* and *CDKN2B*; (B) *CDKN2A* and *ANRIL*; (C) *CDKN2B* and *ANRIL*. Expression values on the X- and Y-axes are shown as delta Ct values for the target gene relative to the three internal control genes. Circles represent individual samples and the crosses represent three outliers excluded from correlation analyses. Linear regression lines are shown as solid lines, with dotted lines indicating the 95% confidence intervals. Pearson correlation coefficient (r) and the P-value for each association are shown in the top left of each plot.
