# Supplementary material for: Chromosome 9p21 SNPs Associated with Multiple Disease Phenotypes Correlate with ANRIL Expression
Source: PLoS Genet. 2010 Apr 8;6(4):e1000899. doi: 10.1371/journal.pgen.1000899 (PMC2851566; doi:10.1371/journal.pgen.1000899)
Supplement: Figure S5 — Correlation between AER in individuals heterozygous for both transcribed markers in a gene. The X- and Y-axes show the allelic expression ratio (AER) at the two transcribed SNPs in each gene. Each point represents an individual who is heterozygous for both transcribed SNPs in that gene. Circles represent CDKN2A, squares CDKN2B, and triangles ANRIL. (0.03 MB DOC) [file pgen.1000899.s005.doc]

**Figure S5. Correlation between AER in individuals heterozygous for both transcribed markers in a gene.** The X- and Y-axes show the allelic expression ratio (AER) at the two transcribed SNPs in each gene. Each point represents an individual who is heterozygous for both transcribed SNPs in that gene. Circles represent *CDKN2A*, squares *CDKN2B*, and triangles *ANRIL*.
