# Supplementary material for: Chromosome 9p21 SNPs Associated with Multiple Disease Phenotypes Correlate with ANRIL Expression
Source: PLoS Genet. 2010 Apr 8;6(4):e1000899. doi: 10.1371/journal.pgen.1000899 (PMC2851566; doi:10.1371/journal.pgen.1000899)
Supplement: Figure S6 — Effect of adjustment for covariates and outliers on total expression mapping. Scatter plots depict the estimates of effect size (A) and significance of association (B) for each of the 56 SNPs obtained using unadjusted total expression values (X-axis) versus values adjusted for covariates (age, sex, ethnicity) and with outliers removed (Y-axis). Pearson correlation coefficient (r) and the P-value for each association are shown in the top left of each plot. (0.05 MB DOC) [file pgen.1000899.s006.doc]

**Figure S6. Effect of adjustment for covariates and outliers on total expression mapping.** Scatter plots depict the estimates of effect size (A) and significance of association (B) for each of the 56 SNPs obtained using unadjusted total expression values (X-axis) versus values adjusted for covariates (age, sex, ethnicity) and with outliers removed (Y-axis). Pearson correlation coefficient (r) and the P-value for each association are shown in the top left of each plot.

r=0.98, P=3x10^-114^

r=0.98, P=2x10^-110^
