# Supplementary material for: Chromosome 9p21 SNPs Associated with Multiple Disease Phenotypes Correlate with ANRIL Expression
Source: PLoS Genet. 2010 Apr 8;6(4):e1000899. doi: 10.1371/journal.pgen.1000899 (PMC2851566; doi:10.1371/journal.pgen.1000899)
Supplement: Figure S7 — Linkage disequilibrium in the SA and Caucasian cohorts. Figures show linkage disequilibrium between the 56 SNPs in each population: (A) D′ in Caucasian cohort; (B) D′ in SA cohort; (C) r2 in Caucasian cohort; (D) r2 in SA cohort. Colouring in (A) and (B) represents D′ values: D′ = 1, LOD<2 (blue); D′ = 1, LOD>2 (red); D′<1, LOD>2 (shades of pink); D′<1, LOD<2 (white). Shading in (C) and (D) represents r2 values: r2 = 1 (black); 0<r2<1 (shades of grey); r2 = 0 (white). (1.05 MB DOC) [file pgen.1000899.s007.doc]

**Figure S7. Linkage disequilibrium in the SA and Caucasian cohorts.** Figures show linkage disequilibrium between the 56 SNPs in each population: (A) D’ in Caucasian cohort; (B) D’ in SA cohort; (C) r^2^ in Caucasian cohort; (D) r^2^ in SA cohort. Colouring in (A) and (B) represents D’ values: D’=1, LOD<2 (blue); D’=1, LOD>2 (red); D’<1, LOD>2 (shades of pink); D’<1, LOD<2 (white). Shading in (C) and (D) represents r^2^ values: r^2^=1 (black); 0< r^2^<1 (shades of grey); r^2^=0 (white).
