# Supplementary material for: Chromosome 9p21 SNPs Associated with Multiple Disease Phenotypes Correlate with ANRIL Expression
Source: PLoS Genet. 2010 Apr 8;6(4):e1000899. doi: 10.1371/journal.pgen.1000899 (PMC2851566; doi:10.1371/journal.pgen.1000899)
Supplement: Figure S8 — Effect of individual normalisation of allelic expression ratios. Scatter plots compare the estimates of effect size (A) and significance of association (B) for each of the 56 SNPs obtained using allelic expression ratios normalised to a combined normalisation factor (X-axis) versus individual normalisation of each cDNA ratio to the gDNA ratio from the same individual (Y-axis). Pearson correlation coefficient (r) and the P-value for each association are shown in the top left of each plot. (0.05 MB DOC) [file pgen.1000899.s008.doc]

**Figure S8. Effect of individual normalisation of allelic expression ratios.** Scatter plots compare the estimates of effect size (A) and significance of association (B) for each of the 56 SNPs obtained using allelic expression ratios normalised to a combined normalisation factor (X-axis) versus individual normalisation of each cDNA ratio to the gDNA ratio from the same individual (Y-axis). Pearson correlation coefficient (r) and the P-value for each association are shown in the top left of each plot.

r=0.995, P=2x10^-164^

r=0.997, P=7x10^-177^
