# Supplementary material for: Chromosome 9p21 SNPs Associated with Multiple Disease Phenotypes Correlate with ANRIL Expression
Source: PLoS Genet. 2010 Apr 8;6(4):e1000899. doi: 10.1371/journal.pgen.1000899 (PMC2851566; doi:10.1371/journal.pgen.1000899)
Supplement: Figure S9 — Linear relationship between measured and expected allelic expression ratios for alleles mixed in known ratios (8∶1, 4∶1, 1∶1, 1∶4, 1∶8) at each transcribed SNP. (A) CDKN2A rs3088440. (B) CDKN2A rs11515. (C) CDKN2B rs3217992. (D) CDKN2B rs1063192. (E) ANRIL rs10965215. (F) ANRIL rs564398. (0.05 MB DOC) [file pgen.1000899.s009.doc]

**Figure S9. Linear relationship between measured and expected allelic expression ratios for alleles mixed in known ratios (8:1, 4:1, 1:1, 1:4, 1:8) at each transcribed SNP.** (A) *CDKN2A* rs3088440. (B) *CDKN2A* rs11515. (C) *CDKN2B* rs3217992. (D) *CDKN2B* rs1063192. (E) *ANRIL* rs10965215. (F) *ANRIL* rs564398.
