# Supplementary material for: Chromosome 9p21 SNPs Associated with Multiple Disease Phenotypes Correlate with ANRIL Expression
Source: PLoS Genet. 2010 Apr 8;6(4):e1000899. doi: 10.1371/journal.pgen.1000899 (PMC2851566; doi:10.1371/journal.pgen.1000899)
Supplement: Figure S10 — Effect of genotype on total expression of ANRIL for selected SNPs. Y-axis shows the normalised total expression value for ANRIL. X-axis shows genotype for SNPs with cis-acting effects: (A) rs564398; (B) rs10965215; (C) rs7865618. Linear regression lines are shown as solid lines, with dotted lines indicating the 95% confidence intervals. (0.10 MB DOC) [file pgen.1000899.s010.doc]

**Figure S10. Effect of genotype on total expression of *ANRIL* for selected SNPs.** Y-axis shows the normalised total expression value for *ANRIL*. X-axis shows genotype for SNPs with *cis*-acting effects: (A) rs564398; (B) rs10965215; (C) rs7865618. Linear regression lines are shown as solid lines, with dotted lines indicating the 95% confidence intervals.
