# Supplementary material for: Chromosome 9p21 SNPs Associated with Multiple Disease Phenotypes Correlate with ANRIL Expression
Source: PLoS Genet. 2010 Apr 8;6(4):e1000899. doi: 10.1371/journal.pgen.1000899 (PMC2851566; doi:10.1371/journal.pgen.1000899)
Supplement: Table S1 — Summary of included SNPs. F = SNP removed from analysis in this cohort as genotype available for <80% of individuals. CAD = coronary artery disease; MAF = minor allele frequency; HW = Hardy-Weinberg. (0.20 MB DOC) [file pgen.1000899.s011.doc]

**Table S1. Summary of included SNPs.** F = SNP removed from analysis in this cohort as genotype available for <80% of individuals. CAD = coronary artery disease; MAF = minor allele frequency; HW = Hardy-Weinberg.

| **SNP ID** | **Chr position** | **Gene info** | **Functional info** | **Selection reason** | **Caucasian cohort** | | | | | **SA cohort** | | | | |
| --- | --- | --- | --- | --- | --- | --- | --- | --- | --- | --- | --- | --- | --- | --- |
|  |  |  |  |  | **% Genotyped** | **MAF** | **Heterozygosity** | **Alleles** | **HW P-value** | **% Genotyped** | **MAF** | **Heterozygosity** | **Alleles** | **HW P-value** |
| rs7023954 | 21806758 | MTAP |  | MTAP gene | 96.0 | 0.44 | 0.50 | G:A | 1.00 | 94.8 | 0.34 | 0.42 | G:A | 0.33 |
| rs15735 | 21852271 | MTAP |  | MTAP gene | 100.0 | 0.25 | 0.44 | A:C | 0.06 | 98.7 | 0.41 | 0.40 | A:C | 0.003 |
| rs1134871 | 21852897 | MTAP |  | MTAP gene | 98.9 | 0.23 | 0.40 | T:A | 0.20 | 100.0 | 0.43 | 0.43 | A:T | 0.04 |
| rs3731257 | 21956221 |  | Ovarian ca | Phenotypic association | 97.7 | 0.32 | 0.39 | G:A | 0.23 | 99.4 | 0.21 | 0.35 | G:A | 0.41 |
| rs3088440 | 21958159 | CDKN2A transcribed | Melanoma, Pancreatic ca, Ovarian ca, Bladder ca | CDKN2A Transcribed | 100.0 | 0.07 | 0.13 | G:A | 1.00 | 100.0 | 0.21 | 0.33 | G:A | 0.87 |
| rs11515 | 21958199 | CDKN2A transcribed | Alzheimers, Bladder ca, Pancreatic ca | CDKN2A transcribed | 98.9 | 0.11 | 0.19 | C:G | 1.00 | 100.0 | 0.14 | 0.24 | C:G | 0.64 |
| rs3731249 | 21960916 |  | Breast ca, melanoma, ALL | Phenotypic association | 98.3 | 0.02 | 0.04 | C:T | 1.00 | 99.7 | 0.01 | 0.02 | C:T | 1.00 |
| rs3731239 | 21964218 |  | CAD, breast ca | Phenotypic association | 95.5 | 0.35 | 0.44 | T:C | 0.73 | 100.0 | 0.15 | 0.26 | T:C | 1.00 |
| rs3814960 | 21965017 | CDKN2A promoter |  | Promoter | F |  |  |  |  | 98.7 | 0.38 | 0.44 | C:T | 0.23 |
| rs36228834 | 21965319 | CDKN2A promoter |  | Promoter | 98.3 | 0.02 | 0.04 | T:A | 1.00 | 99.7 | 0.01 | 0.02 | T:A | 1.00 |
| rs7036656 | 21980457 |  |  | Tag | 96.6 | 0.25 | 0.40 | T:C | 0.56 | 100.0 | 0.23 | 0.36 | T:C | 0.87 |
| rs2811711 | 21983964 | ANRIL promoter |  | Promoter | 98.3 | 0.16 | 0.28 | T:C | 1.00 | 99.4 | 0.09 | 0.16 | T:C | 0.67 |
| rs1801022 | 21984347 | ANRIL promoter |  | Promoter | 97.7 | 0.00 | 0.00 | C:C | 1.00 | 99.7 | 0.00 | 0.00 | C:C | 1.00 |
| rs2518723 | 21985882 | CDKN2A^ARF^ promoter | Colorectal ca | Phenotypic association | 92.1 | 0.43 | 0.41 | C:T | 0.05 | 99.4 | 0.45 | 0.50 | C:T | 0.97 |
| rs3218022 | 21987723 | CDKN2A^ARF^ promoter |  | Promoter | 97.7 | 0.00 | 0.01 | T:C | 1.00 | 99.7 | 0.03 | 0.06 | T:C | 1.00 |
| rs3218020 | 21987872 | CDKN2A^ARF^ promoter |  | Promoter | 100.0 | 0.42 | 0.42 | G:A | 0.08 | 99.7 | 0.25 | 0.40 | G:A | 0.29 |
| rs2811712 | 21988035 | CDKN2A^ARF^ promoter | Frailty, breast ca | Phenotypic association | 97.2 | 0.06 | 0.12 | A:G | 1.00 | 99.7 | 0.22 | 0.37 | A:G | 0.11 |
| rs3218018 | 21988139 | CDKN2A^ARF^ promoter | Diabetes | Phenotypic association | 98.3 | 0.04 | 0.09 | T:G | 1.00 | 99.7 | 0.08 | 0.15 | T:G | 1.00 |
| rs3218012 | 21988660 | CDKN2A^ARF^ promoter | Colorectal ca | Phenotypic association | 97.7 | 0.50 | 0.45 | G:A | 0.18 | 99.7 | 0.46 | 0.53 | G:A | 0.33 |
| rs3218009 | 21988757 | CDKN2A^ARF^ promoter | CAD | Phenotypic association | 96.0 | 0.13 | 0.22 | G:C | 1.00 | 100.0 | 0.03 | 0.06 | G:C | 0.08 |
| rs3218005 | 21990247 |  | Breast ca | Phenotypic association | 96.6 | 0.04 | 0.08 | T:C | 1.00 | 99.4 | 0.21 | 0.36 | T:C | 0.18 |
| rs3217992 | 21993223 | CDKN2B transcribed | CAD | CDKN2B transcribed | 98.3 | 0.46 | 0.41 | G:A | 0.02 | 100.0 | 0.24 | 0.36 | G:A | 1.00 |
| rs1063192 | 21993367 | CDKN2B transcribed | Glioma | CDKN2B transcribed | 98.9 | 0.42 | 0.40 | T:C | 0.02 | 100.0 | 0.18 | 0.28 | T:C | 0.70 |
| rs3217986 | 21995330 | CDKN2B transcribed |  | CDKN2B transcribed | 96.6 | 0.08 | 0.14 | A:C | 0.55 | 100.0 | 0.08 | 0.14 | A:C | 0.64 |
| rs2069418 | 21999698 | CDKN2B promoter |  | Promoter | 97.7 | 0.44 | 0.43 | C:G | 0.15 | 98.7 | 0.18 | 0.30 | C:G | 1.00 |
| rs495490 | 22000412 | RD^INK4/ARF^ |  | Promoter | 95.5 | 0.11 | 0.19 | T:C | 0.69 | 100.0 | 0.03 | 0.05 | T:C | 1.00 |
| rs7044859 | 22008781 |  | CAD, stroke | Phenotypic association | 98.9 | 0.48 | 0.44 | A:T | 0.14 | 100.0 | 0.30 | 0.43 | A:T | 0.97 |
| rs496892 | 22014351 |  | CAD, stroke | Phenotypic association | 100.0 | 0.40 | 0.42 | G:A | 0.14 | 100.0 | 0.30 | 0.45 | G:A | 0.35 |
| rs615552 | 22016077 |  |  | Tag | 95.5 | 0.41 | 0.39 | A:G | 0.02 | 99.7 | 0.17 | 0.29 | A:G | 1.00 |
| rs10965215 | 22019445 | ANRIL transcribed |  | ANRIL transcribed | 100.0 | 0.46 | 0.40 | A:G | 0.01 | 100.0 | 0.42 | 0.50 | G:A | 0.71 |
| rs564398 | 22019547 | ANRIL transcribed | Diabetes, CAD, stroke | ANRIL transcribed | 97.7 | 0.38 | 0.39 | A:G | 0.02 | 100.0 | 0.17 | 0.28 | A:G | 0.99 |
| rs7865618 | 22021005 |  | CAD, stroke | Phenotypic association | 98.9 | 0.41 | 0.39 | A:G | 0.02 | 100.0 | 0.18 | 0.27 | A:G | 0.22 |
| rs17694493 | 22031998 |  |  | Tag | 96.6 | 0.09 | 0.18 | C:G | 0.50 | 100.0 | 0.10 | 0.18 | C:G | 1.00 |
| rs10738605 | 22039130 | ANRIL transcribed |  | ANRIL transcribed | 98.9 | 0.45 | 0.41 | G:C | 0.02 | 99.4 | 0.45 | 0.50 | G:C | 1.00 |
| rs11790231 | 22043591 |  |  | Tag | 96.6 | 0.12 | 0.21 | G:A | 0.96 | 100.0 | 0.06 | 0.11 | G:A | 0.64 |
| rs2184061 | 22051562 |  |  | Tag | 93.2 | 0.38 | 0.41 | A:C | 0.10 | 99.7 | 0.40 | 0.49 | A:C | 0.91 |
| rs1011970 | 22052134 |  | Melanoma | Tag | 83.6 | 0.16 | 0.28 | G:T | 0.96 | 97.4 | 0.28 | 0.42 | G:T | 0.45 |
| rs10811650 | 22057593 |  |  | Tag | 95.5 | 0.47 | 0.37 | C:G | 0.001 | 100.0 | 0.29 | 0.41 | C:G | 1.00 |
| rs16905599 | 22059144 |  |  | Tag | F |  |  |  |  | 91.9 | 0.19 | 0.29 | G:A | 0.51 |
| rs10116277 | 22071397 |  | CAD, stroke | Phenotypic association | 100.0 | 0.49 | 0.36 | T:G | 0.0002 | 100.0 | 0.26 | 0.37 | T:G | 0.63 |
| rs10965227 | 22071796 |  |  | Tag | 96.0 | 0.22 | 0.35 | A:G | 1.00 | 100.0 | 0.09 | 0.15 | A:G | 0.20 |
| rs1547705 | 22072375 |  |  | Tag | 96.0 | 0.12 | 0.19 | A:C | 0.75 | 99.7 | 0.11 | 0.19 | A:C | 0.95 |
| rs10965228 | 22072380 |  |  | Tag | 96.6 | 0.12 | 0.21 | A:G | 0.96 | 100.0 | 0.03 | 0.06 | A:G | 1.00 |
| rs1333040 | 22073404 |  | CAD, stroke | Phenotypic association | 100.0 | 0.36 | 0.37 | T:C | 0.02 | 99.4 | 0.41 | 0.48 | T:C | 0.94 |
| rs7857345 | 22077473 |  |  | Tag | 93.2 | 0.27 | 0.39 | C:T | 1.00 | 100.0 | 0.14 | 0.21 | C:T | 0.02 |
| rs10757274 | 22086055 |  | CAD | Phenotypic association | 100.0 | 0.49 | 0.40 | G:A | 0.01 | 100.0 | 0.38 | 0.45 | A:G | 0.50 |
| rs10125231 | 22092128 |  |  | Tag | 94.9 | 0.02 | 0.03 | G:A | 1.00 | 100.0 | 0.01 | 0.02 | G:A | 1.00 |
| rs2383206 | 22105026 |  | CAD, stroke | Phenotypic association | 100.0 | 0.47 | 0.40 | G:A | 0.01 | 100.0 | 0.48 | 0.51 | G:A | 0.81 |
| rs2383207 | 22105959 |  | CAD, stroke | Phenotypic association | 90.4 | 0.42 | 0.44 | G:A | 0.33 | 100.0 | 0.23 | 0.32 | G:A | 0.15 |
| rs1333045 | 22109195 |  | CAD | Tag | 96.6 | 0.48 | 0.42 | C:T | 0.05 | 99.7 | 0.49 | 0.51 | T:C | 0.97 |
| rs10757278 | 22114477 |  | CAD, stroke | Phenotypic association | 99.4 | 0.50 | 0.42 | A:A | 0.05 | 100.0 | 0.36 | 0.41 | A:G | 0.05 |
| rs1333049 | 22115503 |  | CAD | Phenotypic association | 100.0 | 0.50 | 0.43 | C:G | 0.08 | 100.0 | 0.38 | 0.45 | G:C | 0.41 |
| rs2891169 | 22121825 |  | Diabetes | Phenotypic association | F |  |  |  |  | 99.4 | 0.48 | 0.49 | G:A | 0.92 |
| rs2383208 | 22122076 |  | Diabetes | Phenotypic association | 93.2 | 0.18 | 0.29 | A:G | 0.84 | 99.4 | 0.24 | 0.35 | A:G | 0.40 |
| rs10811661 | 22124094 |  | Diabetes | Phenotypic association | 100.0 | 0.17 | 0.28 | T:C | 0.84 | 99.7 | 0.11 | 0.19 | T:C | 0.61 |
| rs10757283 | 22124172 |  | Diabetes | Phenotypic association | 97.7 | 0.47 | 0.42 | C:T | 0.06 | 99.7 | 0.49 | 0.49 | C:T | 0.76 |
