# Supplementary material for: Chromosome 9p21 SNPs Associated with Multiple Disease Phenotypes Correlate with ANRIL Expression
Source: PLoS Genet. 2010 Apr 8;6(4):e1000899. doi: 10.1371/journal.pgen.1000899 (PMC2851566; doi:10.1371/journal.pgen.1000899)
Supplement: Table S2 — Effect size and significance of association for SNPs associated with disease. Data shown are for aeQTL mapping in the combined population. Effects are reported as fold changes in expression for individuals who are homozygous for the minor allele relative to individuals who are homozygous for the major allele (calculated from allelic expression data using two transcribed SNPs per gene). Association for each SNP is presented as the −log10 P-value and the −log10 of the family wise error rate (FWER) using a Bonferroni correction for the 56 SNPs tested. Associations that were significant using a FWER threshold of 0.05 (corresponding to −log10P of 3.05, or −log10FWER of 1.3) were regarded as significant. (0.20 MB DOC) [file pgen.1000899.s012.doc]

**Table S2. Effect size and significance of association for all SNPs.**

| **SNP** | **Promoter** | **Reported phenotypic associations** | **Risk allele** | **Minor allele** | ***CDKN2A* effect**  **(fold change)** | ***CDKN2A***  **-log_10_P** | ***CDKN2A***  **-log_10_FWER** | ***CDKN2B* effect**  **(fold change)** | ***CDKN2B***  **-log_10_P** | ***CDKN2B***  **-log_10_FWER** | ***ANRIL* effect (fold change)** | ***ANRIL***  **–log_10_P** | ***ANRIL***  **-log_10_FWER** |
| --- | --- | --- | --- | --- | --- | --- | --- | --- | --- | --- | --- | --- | --- |
| **rs7023954** |  |  |  | A | 0.989 | 0.2 | 0 | 1.000 | 0 | 0 | 0.949 | 0.5 | 0 |
| **rs15735** |  |  |  | C | 1.026 | 0.5 | 0 | 1.003 | 0.1 | 0 | 1.040 | 0.3 | 0 |
| **rs1134871** |  |  |  | A | 0.997 | 0 | 0 | 1.038 | 2 | 0.3 | 1.057 | 0.5 | 0 |
| **rs3731257** |  | Ovarian ca | G | A | 0.883 | 3.8 | 2.1 | 1.011 | 0.4 | 0 | 0.596 | 24.1 | 22.4 |
| **rs3088440** |  | Melanoma, pancreatic ca, ovarian ca, bladder ca | A | A | 0.919 | 3.8 | 2.1 | 1.050 | 1.8 | 0.1 | 0.828 | 2.1 | 0.4 |
| **rs11515** |  | Alzheimers, bladder ca, pancreatic ca | C | G | 1.084 | 4.7 | 3.0 | 0.880 | 8.7 | 7.0 | 0.968 | 0.2 | 0 |
| **rs3731249** |  | Breast ca, melanoma, ALL | T | T | 1.404 | 7.5 | 5.8 | 0.752 | 4.1 | 2.4 | 0.899 | 0.1 | 0 |
| **rs3731239** |  | CAD, breast ca | A | C | 0.947 | 1 | 0 | 0.997 | 0.1 | 0 | 1.652 | 25.2 | 23.5 |
| **rs3814960** | CDKN2A |  |  | T | 0.898 | 3.7 | 2.0 | 0.988 | 0.4 | 0 | 1.039 | 0.3 | 0 |
| **rs36228834** | CDKN2A |  |  | A | 1.404 | 7.5 | 5.8 | 0.752 | 4.1 | 2.4 | 0.899 | 0.1 | 0 |
| **rs7036656** |  |  |  | C | 1.119 | 8.7 | 7.0 | 0.931 | 4.9 | 3.2 | 1.217 | 3.1 | 1.4 |
| **rs2811711** | ANRIL |  |  | C | 1.197 | 4.2 | 2.5 | 0.965 | 1.1 | 0 | 1.099 | 0.7 | 0 |
| **rs1801022** | ANRIL |  |  | C | NA | NA | NA | NA | NA | NA | NA | NA | NA |
| **rs2518723** | CDKN2A/ARF | Colorectal ca promoter methylation | C | T | 1.119 | 6.1 | 4.4 | 0.971 | 2 | 0.3 | 1.669 | 51.5 | 49.8 |
| **rs3218022** | CDKN2A/ARF |  |  | C | 1.045 | 0.2 | 0 | 0.985 | 0.1 | 0 | 0.995 | 0 | 0 |
| **rs3218020** | CDKN2A/ARF |  |  | A | 0.989 | 0.2 | 0 | 1.008 | 0.3 | 0 | 0.587 | 38.2 | 36.5 |
| **rs2811712** | CDKN2A/ARF | Frailty, breast ca | A | G | 1.079 | 2.9 | 1.2 | 0.938 | 3.1 | 1.4 | 1.155 | 1.5 | 0 |
| **rs3218018** | CDKN2A/ARF | Diabetes | G | G | 1.108 | 4 | 2.3 | 0.805 | 14.5 | 12.8 | 1.097 | 0.4 | 0 |
| **rs3218012** | CDKN2A/ARF | Colorectal ca promoter methylation | A | A | 0.919 | 3.8 | 2.1 | 1.026 | 1.8 | 0.1 | 0.605 | 53.1 | 51.4 |
| **rs3218009** | CDKN2A/ARF | CAD | G | C | 1.059 | 0.4 | 0 | 0.990 | 0.2 | 0 | 1.659 | 7.7 | 6.0 |
| **rs3218005** |  | Breast ca, frailty | C | C | 1.087 | 3.2 | 1.5 | 0.940 | 2.7 | 1.0 | 1.163 | 1.5 | 0 |
| **rs3217992** |  | CAD | A | A | 0.990 | 0.1 | 0 | 1.009 | 0.4 | 0 | 0.579 | 39.7 | 38.0 |
| **rs1063192** |  | Glioma | C | C | 1.036 | 0.5 | 0 | 0.976 | 1.4 | 0 | 1.829 | 61.3 | 59.6 |
| **rs3217986** |  |  |  | C | 0.914 | 2.2 | 0.5 | 1.049 | 1.1 | 0 | 0.605 | 8.3 | 6.6 |
| **rs2069418** | CDKN2B |  |  | G | 1.031 | 0.5 | 0 | 0.976 | 1.4 | 0 | 1.852 | 72 | 70.3 |
| **rs495490** | RD^INK4/ARF^ |  |  | C | 1.131 | 1.1 | 0 | 0.955 | 1.2 | 0 | 1.970 | 12.8 | 11.1 |
| **rs7044859** |  | CAD, stroke | A | T | 1.089 | 3.6 | 1.9 | 0.945 | 6.4 | 4.7 | 1.797 | 61.7 | 60.0 |
| **rs496892** |  | CAD, stroke | G | A | 1.102 | 4.5 | 2.8 | 0.952 | 4.7 | 3.0 | 1.775 | 63.3 | 61.6 |
| **rs615552** |  |  |  | G | 1.028 | 0.4 | 0 | 0.980 | 1 | 0 | 1.857 | 80 | 78.3 |
| **rs10965215** |  |  |  | A | 0.936 | 2.1 | 0.4 | 1.014 | 0.7 | 0 | 0.599 | 76.6 | 74.9 |
| **rs564398** |  | Diabetes, CAD, stroke | A | G | 1.034 | 0.5 | 0 | 0.978 | 1.2 | 0 | 1.865 | 81.3 | 79.6 |
| **rs7865618** |  | CAD, stroke | A | G | 1.035 | 0.5 | 0 | 0.979 | 1.1 | 0 | 1.870 | 78.2 | 76.5 |
| **rs17694493** |  |  |  | G | 1.103 | 3.5 | 1.8 | 0.862 | 10.2 | 8.5 | 1.234 | 1.9 | 0.2 |
| **rs10738605** |  |  |  | C | 1.116 | 5.6 | 3.9 | 0.968 | 2.4 | 0.7 | 1.689 | 57.5 | 55.8 |
| **rs11790231** |  |  |  | A | 1.006 | 0 | 0 | 0.980 | 0.4 | 0 | 0.618 | 7.1 | 5.4 |
| **rs2184061** |  |  |  | C | 1.021 | 0.4 | 0 | 1.010 | 0.4 | 0 | 1.697 | 53.2 | 51.5 |
| **rs1011970** |  | Melanoma | T | T | 0.995 | 0.1 | 0 | 0.953 | 2.5 | 0.8 | 0.802 | 3.8 | 2.1 |
| **rs10811650** |  |  |  | G | 0.988 | 0.2 | 0 | 1.000 | 0 | 0 | 0.629 | 31.7 | 30.0 |
| **rs16905599** |  |  |  | A | 0.907 | 3.6 | 1.9 | 1.028 | 0.7 | 0 | 0.684 | 8.1 | 6.4 |
| **rs10116277** |  | CAD, stroke | T | G | 0.979 | 0.4 | 0 | 0.984 | 0.7 | 0 | 1.573 | 21.9 | 20.2 |
| **rs10965227** |  |  |  | G | 0.966 | 0.5 | 0 | 0.949 | 2.2 | 0.5 | 1.008 | 0 | 0 |
| **rs1547705** |  |  |  | C | 1.031 | 0.4 | 0 | 0.999 | 0 | 0 | 0.734 | 3.6 | 1.9 |
| **rs10965228** |  |  |  | G | 1.104 | 0.8 | 0 | 0.958 | 1.2 | 0 | 1.504 | 6 | 4.3 |
| **rs1333040** |  | CAD, stroke | T | C | 0.963 | 1 | 0 | 1.012 | 0.4 | 0 | 1.406 | 12.1 | 10.4 |
| **rs7857345** |  |  |  | T | 0.935 | 1.3 | 0 | 1.016 | 0.5 | 0 | 1.779 | 25 | 23.3 |
| **rs10757274** |  | CAD | G | G | 1.039 | 1 | 0 | 0.975 | 1.2 | 0 | 0.685 | 14.5 | 12.8 |
| **rs10125231** |  |  |  | A | 1.101 | 0.3 | 0 | 0.961 | 0.3 | 0 | 1.280 | 0.7 | 0 |
| **rs2383206** |  | CAD, stroke | G | A | 0.968 | 0.9 | 0 | 1.007 | 0.2 | 0 | 1.456 | 15 | 13.3 |
| **rs2383207** |  | CAD, stroke | G | A | 0.946 | 1.5 | 0 | 0.982 | 0.7 | 0 | 1.468 | 14 | 12.3 |
| **rs1333045** |  | CAD | C | C | 1.037 | 1 | 0 | 0.981 | 0.8 | 0 | 0.700 | 12.8 | 11.1 |
| **rs10757278** |  | CAD, stroke | G | G | 1.039 | 0.9 | 0 | 0.986 | 0.5 | 0 | 0.700 | 11.7 | 10.0 |
| **rs1333049** |  | CAD | C | C | 1.023 | 0.5 | 0 | 0.996 | 0.1 | 0 | 0.704 | 12.1 | 10.4 |
| **rs2891169** |  | Diabetes | G | A | 1.042 | 1 | 0 | 0.977 | 0.8 | 0 | 1.059 | 0.5 | 0 |
| **rs2383208** |  | Diabetes | G | G | 1.081 | 1.9 | 0.2 | 0.989 | 0.3 | 0 | 1.239 | 3.4 | 1.7 |
| **rs10811661** |  | Diabetes | T | C | 1.182 | 4 | 2.3 | 0.957 | 1.8 | 0.1 | 1.339 | 4.6 | 2.9 |
| **rs10757283** |  | Diabetes | T | T | 1.010 | 0.2 | 0 | 1.002 | 0.1 | 0 | 1.024 | 0.2 | 0 |

Data shown are for aeQTL mapping in the combined population. Effects are reported as fold changes in expression for individuals who are homozygous for the minor allele relative to individuals who are homozygous for the major allele (calculated from allelic expression data using two transcribed SNPs per gene). Association for each SNP is presented as the –log_10_ P-value and the –log_10_ of the family wise error rate (FWER) using a Bonferroni correction for the 56 SNPs tested. Associations that were significant using a FWER threshold of 0.05 (corresponding to –log_10_P of 3.05, or –log_10_FWER of 1.3) were regarded as significant.
