# Supplementary material for: Chromosome 9p21 SNPs Associated with Multiple Disease Phenotypes Correlate with ANRIL Expression
Source: PLoS Genet. 2010 Apr 8;6(4):e1000899. doi: 10.1371/journal.pgen.1000899 (PMC2851566; doi:10.1371/journal.pgen.1000899)
Supplement: Table S4 — Comparison of allelic expression normalisation ratios obtained from genomic DNA and experimental equimolar mixtures. (0.03 MB DOC) [file pgen.1000899.s014.doc]

**Table S4. Comparison of allelic expression normalisation ratios obtained from genomic DNA and experimental equimolar mixtures.**

| **Gene** | **SNP** | **Ratio** | **Genomic DNA** | **Equimolar mix** |
| --- | --- | --- | --- | --- |
| ***CDKN2A*** | rs3088440 | G/A | 0.87 | 0.82 |
|  | rs11515 | C/G | 1.26 | 1.36 |
| ***CDKN2B*** | rs3217992 | G/A | 0.97 | 1.08 |
|  | rs1063192 | C/T | 0.96 | 1.01 |
| ***ANRIL*** | rs10965215 | G/A | 0.97 | 1.02 |
|  | rs564398 | G/A | 0.81 | 0.83 |
