# Supplementary material for: Chromosome 9p21 SNPs Associated with Multiple Disease Phenotypes Correlate with ANRIL Expression
Source: PLoS Genet. 2010 Apr 8;6(4):e1000899. doi: 10.1371/journal.pgen.1000899 (PMC2851566; doi:10.1371/journal.pgen.1000899)
Supplement: Table S5 — Comparison of variances between total expression and allelic expression measurements in the SA cohort. (0.03 MB DOC) [file pgen.1000899.s015.doc]

**Table S5. Comparison of variances between total expression and allelic expression measurements in the SA cohort.**

| Total expression | | | | Allelic expression | | | |
| --- | --- | --- | --- | --- | --- | --- | --- |
| Gene | Variance within samples | Standard error within samples | Variance between samples | Transcribed marker | Variance within samples | Standard error within samples | Variance between samples |
| *CDKN2A* | 0.057 | 0.11 | 0.434 | rs3088440 | 0.016 | 0.06 | 0.111 |
|  |  |  |  | rs11515 | 0.028 | 0.07 | 0.064 |
| *CDKN2B* | 0.038 | 0.08 | 0.478 | rs1063192 | 0.043 | 0.05 | 0.044 |
|  |  |  |  | rs3217992 | 0.018 | 0.04 | 0.039 |
| *ANRIL* | 0.590 | 0.31 | 0.697 | rs10965215 | 0.073 | 0.12 | 0.106 |
|  |  |  |  | rs564398 | 0.063 | 0.11 | 0.076 |
